# The influence of n-3 polyunsaturated fatty acids on cognitive function in individuals without dementia: a systematic review and dose–response meta-analysis

**DOI:** 10.1186/s12916-024-03296-0

**Published:** 2024-03-12

**Authors:** Seung Wan Suh, Eunji Lim, Suh-Yuhn Burm, Hyungji Lee, Jong Bin Bae, Ji Won Han, Ki Woong Kim

**Affiliations:** 1 Seoul Heal Mental Health Clinic, Seoul, South Korea; 2 Department of Psychiatry, Gyeongsang National University Changwon Hospital, Changwon, South Korea; 3 Department of Neuropsychiatry, Seoul National University Bundang Hospital, Seongnam, South Korea; 4 Department of Psychiatry, Seoul National University, College of Medicine, Seoul, South Korea; 5 Department of Brain and Cognitive Sciences, Seoul National University, College of Natural Sciences, Seoul, South Korea

**Keywords:** N-3 PUFA, Polyunsaturated fatty acids, Omega-3, Mild cognitive impairment, Cognitive function, Dose–response meta-analysis

## Abstract

**Background:**

Omega-3 polyunsaturated fatty acids (n-3 PUFA) have been suggested as a cognitive enhancing agent, though their effect is doubtful. We aimed to examine the effect of n-3 PUFA on the cognitive function of middle-aged or older adults without dementia.

**Methods:**

We reviewed randomized controlled trials of individuals aged 40 years or older. We systematically searched PubMed/MEDLINE, EMBASE, CINAHL, PsycINFO, and Cochrane Library databases. We used the restricted cubic splines model for non-linear dose–response meta-analysis in terms of the standardized mean difference with 95% confidence intervals.

**Results:**

The current meta-analysis on 24 studies (*n* 9660; follow-up 3 to 36 months) found that the beneficial effect on executive function demonstrates an upward trend within the initial 12 months of intervention. This effect is prominently observed with a daily intake surpassing 500 mg of n-3 PUFA and up to 420 mg of eicosapentaenoic acid (EPA). Furthermore, these trends exhibit heightened significance in regions where the levels of blood docosahexaenoic acid (DHA) + EPA are not very low.

**Conclusions:**

Supplementation of n-3 PUFA may confer potential benefits to executive function among the middle-aged and elderly demographic, particularly in individuals whose dietary DHA + EPA level is not substantially diminished.

**Supplementary Information:**

The online version contains supplementary material available at 10.1186/s12916-024-03296-0.

## Background

In 1971, Bang et al. [1] suggested a beneficial effect of the dietary n-3 polyunsaturated fatty acid (PUFA) such as docosahexaenoic acid (DHA) or eicosapentaenoic acid (EPA) on the incidence of ischemic heart disease. Since then, abundant epidemiological studies have explored its role on the cognitive function in human [2–4] under the assumption that the n-3 PUFA helps resolve inflammation and facilitate brain development [5], reduces accumulation of amyloid β [6], and increases the production of brain-derived neurotrophic factor (BDNF) [7].

Unfortunately, the efficacy of n-3 PUFAs on cognition has not been proven consistently in previous meta-analyses. In older adults aged 60 years or older with mild cognitive impairment (MCI), n-3 PUFAs were beneficial on mini-mental status examination (MMSE) (weighted mean difference = 0.85) [8]. Conversely, in adults aged 18 years or older, the impact of n-3 PUFA on MMSE was either negligible [9] or demonstrated very modest benefits at best resulting in less than 1% change in MMSE score [10]. Moreover, in younger adults aged between their 20 s and 30 s, no discernible influence of n-3 PUFA on cognition was identified [11, 12]. McCaddon and Miller asserted that to have any expectation of observing an effect of a nutritional intervention within a typical time frame of clinical trials from months to years, it is required that the participants should experience cognitive decline or on the verge of it during the study period [13]. These findings suggest that the inclusion of young adults in trials is unlikely to yield a significant effect of the intervention material on age-related cognitive trajectories [14]. Given that this age-related cognitive decline might commence as early as in 20 s to 30 s [15], it would be judicious to restrict the inclusion criteria to individuals aged 40 or older for meta-analyses. On the other hand, MCI represents a syndrome distinguished by cognitive impairment that deviates from the anticipated age-related trajectories, while daily functioning is not disturbed to qualify for the diagnosis of dementia [16]. Therefore, it would be meaningful to investigate the potential impact of n-3 PUFA with respect to the presence of MCI among the included studies.

Previous meta-analyses have primarily investigated the relationship between dose and response using linear models on subsets of included trials. However, this linear relationship could rarely be presumed in biological research [17], and subgroup analyses by categorizing trials could lead to a reduction in power and loss of information pertaining to the association to be examined [18, 19]. In this regard, the application of spline functions, which employ all available data points to explore the dose–response relationship between the intervention and the outcome, is capable of modeling intricate nonlinear associations [20, 21]. While several previous dose–response meta-analyses have already been conducted, they have either included, in their analyses, very young adults around the age of 30 [22], exclusively dealt with prospective cohort studies [23, 24], or were unable to conduct dose–response analyses due to data scarcity [8]. Furthermore, none of the preceding meta-analyses have investigated this association in relation to the nationwide blood levels of DHA + EPA, despite the potential influence of early-life or long-term exposure to n-3 PUFA on cognitive function [25].

Therefore, the objective of this study was to examine the impact of n-3 PUFAs on cognitive function in non-demented individuals, encompassing both cognitively normal older adults and those with MCI, belonging to the middle-aged or older age group (≥ 40 years old). This investigation employed restricted cubic splines models [26] on randomized controlled trials with subgroup analyses by the nation-wide blood level of DHA + EPA and by the presence of MCI.

## Methods

This study was conducted following the Preferred Reporting Items for Systematic Reviews and Meta-Analyses (PRISMA) guidelines [27] and its protocol was registered on the International Prospective Register of Systematic Reviews (PROSPERO, registration number: CRD42020221943).

### Criteria for study inclusion/exclusion

The selection of studies based on the Population, Intervention, Comparison, Outcomes, and Study Design (PICOS) criteria for this review is as follows: (1) inclusion of human subjects without dementia, aged 40 years or older; (2) incorporation of interventions involving n-3 PUFA, DHA, EPA, or alpha-linolenic acid (ALA); (3) administration of intervention supplements alone or in combination with other supplements, excluding B vitamins; (4) implementation of interventions lasting 3 or more months; (5) outcomes in the form of cognitive test scores; (6) availability of the mean difference of the test scores before and after the intervention, along with dispersion data such as standard deviation (SD), standard errors of the mean (SEM), confidence intervals, *t* statistics, *P* values, or *F* statistics; (7) structured as a randomized placebo-controlled clinical trial; and (8) studies published in any language.

### Search strategy and study selection

Electronic searches of the PubMed/MEDLINE, EMBASE, CINAHL, PsycINFO, and Cochrane Library database were performed by SW Suh, E Lim, and KW Kim from inception to Sep 2023. Searches for the gray literature were also sought through the International Clinical Trials Registry Platform Search Portal. Search strategies for each database are presented in the Additional file 1: Table S1 which were built based on a previous literature.

From the search results, S-Y Burm and H Lee selected studies independently that fit the inclusion and exclusion criteria based on their titles followed by their abstracts. Subsequent full-text evaluation of the selected studies was conducted independently by SW Suh and E Lim. Non-English texts were translated into English using Google Translate. Disagreement of the selection results between two investigators that are not resolved by their discussion was settled by KW Kim and JW Han.

### Outcomes

For the primary outcome, we used the standardized mean difference of the test scores on global cognitive function between baseline and follow-up assessments. For the secondary outcomes, we used the mean difference of the test scores on episodic memory, executive function, processing speed, attention, and visuospatial function between baseline and follow-up.

### Data extraction and assessment of methodological quality

SW Suh and E Lim, working independently, extracted data on the study design, recruitment setting and location, sample size, baseline characteristics of participants such as age and sex ratio, intervention methods (ingredients, dosage, frequency, and duration), compliance to the intervention, funding sources, and the mean difference in the cognitive performance of participants before and after the intervention along with its corresponding SD or SEM. The mean difference was represented as standardized mean difference (SMD), factoring in the combined SD, with correction for small sample bias using Hedges’ *g* [28]. In instances where the dispersion data on the mean difference was not provided, it was computed in adherence to the Cochrane guidelines [29]. We grouped the cognitive tests by the cognitive domain they mainly represent based on a previous work [30]. If a study reported outcomes of multiple tests for a single cognitive domain, we chose the most frequently used test among the overall included studies to maximize the homogeneity of the outcome variable. If there were several scores of multiple time points for a given cognitive test under random-effects model, we used the one with the longest intervention period. Additionally, in case that a given study did not report numerical data of cognitive test scores, we approximated the means and measures of dispersion from figures, if available. For studies with a crossover design, we only used data prior to the crossover.

SW Suh and E Lim also evaluated risk of bias (RoB) of each included study using the RoB Tool from the Cochrane Handbook [29] in respect of the random sequence generation, allocation concealment, blinding of participants and personnel, blinding of outcome assessment, incomplete outcome data, selective reporting, and other biases. We made summary assessments of the RoB by the order of priority of the following rules: (1) “high” for the high risk of one or more key domains; (2) “unclear” for the unclear risk of one or more key domains; and (3) “low” for the low risk of all key domains [29]. We determined the key domains to be the allocation concealment, blinding of participants and personnel, and blinding of outcome assessment because the outcome, cognitive test scores, could be critically affected by the preconception of the interviewer and/or interviewee. Disagreement between these two investigators about the extracted data or RoB that was not resolved by a discussion was settled by KW Kim and JW Han. We requested necessary information that was not available in the published article by e-mailing to the respective corresponding author.

### Statistical analyses

We first synthesized data using standard inverse-variance random-effects model [31] for meta-analyses utilizing standardized mean differences (SMD) along with their corresponding 95% confidence intervals (CIs). This approach was adopted due to the diverse outcome measures employed by researchers in assessing cognitive function. We also conducted sensitivity analyses by eliminating one study at a time to examine the influence of each study on the results. We assessed the between-trial heterogeneity using *I*^2^ and *τ*^2^ values. Publication bias was ascertained by Egger’s test when at least 7 studies were included, as well as the visual inspection of the funnel plot.

For the nonlinear dose–response meta-analysis, we used a restricted cubic spline model applying weighted mixed-effects models [32] in accordance with the methodology demonstrated by Orsini et al. [26] with 3 knots at fixed percentiles (5, 50, and 95%). Estimates of the mixed-effects model were acquired by a restricted maximum likelihood method [32]. Through this, we evaluate the impact of key variables including the duration of the intervention (measured in months), daily intake of n-3 PUFA (in milligrams per day), cumulative PUFA consumption over the study period (in grams), daily intake of DHA (in milligrams per day), daily intake of EPA (in milligrams per day), and the ratio of DHA to EPA consumed. To ascertain nonlinearity, we conducted a Wald test [33]. In addition, we executed a linear dose–response meta-analysis, adhering to the approach elucidated by Greenland and Longnecker [34] and compared the goodness of fit, denoted by *χ*^2^, between the nonlinear and linear models. Furthermore, we visually examined the plot to facilitate the interpretation of the corresponding curve.

We performed a priori subgroup analyses for the primary outcome and for the secondary outcomes with significant nonlinear associations in the main analyses (1) by the nation-wide blood level of DHA + EPA (very low level with ≤ 4% such as USA, UK, Ireland, and Italy versus other countries with > 4% in erythrocyte equivalents) [35] and (2) by baseline cognitive function (normal cognition vs MCI). The blood level was defined as the percentage of DHA + EPA of total fatty acids in erythrocyte equivalents. All statistical analyses were executed using the R statistical software (version 4.3.1; R Foundation for Statistical Computing, Vienna, Austria) with a *P*-value of < 0.05 set as a statistical significance.

## Results

### Study selection

**Fig. 1:**
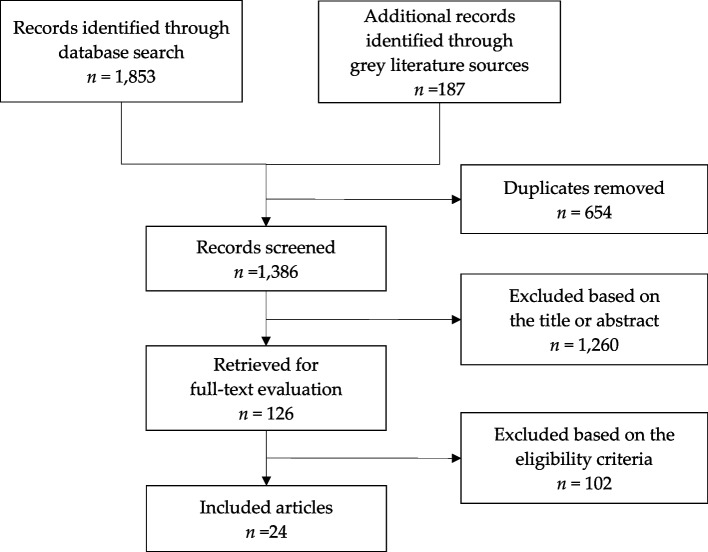
Preferred Reporting Items for Systematic Reviews and Meta-Analysis (PRISMA) diagram

We retrieved 2040 articles and excluded 654 duplicates. Of the remaining 1386 articles, we excluded 1260 articles by screening the titles and abstracts. At this stage, we came to exclude all the non-English articles. Of the remaining 126 articles, we finally included 24 articles in the current systematic review after excluding 102 articles by full-text evaluation; 38 were not randomized or placebo-controlled, 11 had the intervention duration of less than 3 months, 17 included the participants under 40 years old, 10 included subjects who participated in other included studies, and 26 did not provide appropriate cognitive test results. We reached out to the corresponding authors but failed to get additional information on the missing data (Fig. 1).

### Study characteristics

The characteristics of 24 individual trials are presented in Table 1. The sample size of them ranged from 29 to 2461, and the number of participants included in the current meta-analysis was 9660. Nine studies included the subjects with normal cognition only [36–44] while four studies composed only of people with MCI [45–48]. As for the intervention, three studies employed DHA only [36, 44, 49] and four studies employed ALA only [37, 39, 43, 50]. The length of intervention ranged from 3 to 36 months, and the daily dose of n-3 PUFA ranged from 230 to 4000 mg/day. Six studies were conducted in countries where the nationwide blood levels of DHA + EPA were notably low, measuring ≤ 4% in erythrocyte equivalents [36, 40, 44, 50–52].

**Table 1:** Characteristics of included studies

Study (year)	Country	Group	Age Mean (SD)	Female (%)	N	Duration (month)	n-3 PUFA (mg/day)	Cognitive measures used	Corresponding domain	Notes	Summary RoB
Andrieu (2017) [53]	France	DHA, EPA	75.6 (4.7)	64	320	36	1025	MMSE, FCSRT, CNT, DSST, TMT-A	GLO, MEM, EF, PS, ATT		Low
		Placebo	75.1 (4.3)	66	325						
Arellanes (2020) [36]	USA	DHA	68.5 (58,90)^*^	89	15	6	2152	MoCA, CVLT, TMT-B, TMT-A	GLO, MEM, EF, ATT	CN at baseline	Unclear
		Placebo	69 (58,79)^*^	73	14						
Bischoff-Ferrari (2020) [54]	Europe	DHA, EPA	74.7 (4.3)	62.3	1073	12, 24, 36	990	MoCA	GLO		Low
		Placebo	75.2 (4.6)	61.2	1084						
Bo (2017) [45]	China	DHA, EPA	71.8 (5.68)	40.9	44	6	1200	BCAT, RM, MAE, PCT, SIE	GLO, MEM, EF, PS, VSF	MCI at baseline	Unclear
		Placebo	70.5 (6.82)	40.5	42						
Chew (2015) [51]	USA	DHA, EPA	72.7 (7.7)	58.8	1226	12	1000	TICS	GLO		Low
		Placebo	72.7 (7.8)	56.2	1235						
Dadova (2022) [55]	Czech Republic	DHA, EPA	70.8 (3.6)	100	27	4	230	POBAV	MEM	Only women	Unclear
		Placebo	71.2 (4.0)	100	25						
Dangour (2010) [52]	UK	DHA, EPA	74.7 (2.5)	46.6	376	24	700	GCF, CVLT, VF, SLM, DSF	GLO, MEM, EF, PS, ATT		Low
		Placebo	74.6 (2.7)	43.4	372						
Geleijnse (2012) [56]	Netherlands	DHA, EPA	69.2 (5.4)	33	627	10	400	MMSE	GLO		Low
		Placebo	68.9 (5.4)	20	638						
Hashimoto (2016) [49]	Japan	DHA	87.6 (3.3)	88.4	39	6, 12	1720	MMSE	GLO		Unclear
		Placebo	89.6 (5.1)	84.4	27						
Hashimoto (2021) [37]	Japan	ALA	71 (65, 83)^*^	54.8	35	12	4000	FAB	EF	CN at baseline	High
		Placebo	71 (64, 84)^*^	51.5	24						
Howe (2018) [57]	Australia	DHA, EPA	63.2 (1.6)	31.6	19	5	2000	Corresponding *Z* scores (See Table S2)	GLO, EF, PS	Borderline hypertensive adults	Low
		Placebo	64.1 (2.3)	31.6	19						
Ichinose (2021) [38]	Japan	DHA, EPA	69.1 (5.3)	53.8	26	12	434 (297, 137)	MMSE	GLO	CN at baseline	Low
		Placebo	69.1 (4.9)	55.6	27						
Kuszewski (2020) [58]	Australia	DHA, EPA	65.8 (1.4)	53	26	4	2400	RAVLT, Cognitive flexibility	MEM, EF		Low
		Placebo	65.8 (1.4)	56	26						
Lee (2013) [46]	Malaysia	DHA, EPA	66.4 (5.1)	82.4	17	6, 12	1750	Corresponding* Z* scores (see Table S2)	MEM, EF, PS, VSF	MCI at baseline	Low
		Placebo	63.5 (3.0)	72.2	18						
Macpherson (2022) [59]	Australia	DHA, EPA	70.0 (6.3)	70	69	6	1500	MoCA, TMT-B, CPF-z, TMT-A	GLO, EF, PS, ATT	Intervened only 6 months. Next 6 months was without n-3 PUFA	Low
		Placebo	70.5 (5.9)	70	72						
Mengelberg (2022) [47]	New Zealand	DHA, EPA	72.3 (6.2)	53	30	12	1842	RTot, RDM, TMT-B, DSF, RVC	GLO, MEM, EF, ATT, VSF		Low
		Placebo	734. (7.0)	63	30						
Ogawa (2023) [39]	Japan	ALA	71.9 (3.7)	46.7	30	3	2200	MMSE, WMSR-VM, LF, WAIS-PSI, WMSR-AC, WAIS-PRI	GLO, MEM, EF, PS, ATT, VSF		Low
		Placebo	72.1 (4.3)	53.3	30						
Power (2022) [40]	Ireland	DHA, EPA	69.0 (4.4)	56.7	28	24	520	RTot, SWM, SRT,	GLO, EF, PS	CN at baseline	Low
		Placebo	69.8 (3.7)	70.0	22						
Sala-Vila (2020) [50]	USA	ALA	69.4 (3.8)	67.4	324	6	1206	RAVLT, SF, SDMT, TMT-A, block design	MEM, EF, PS, ATT, VSF	Constituents were determined by FoodData Central^**^ CN at baseline	High
		Placebo	68.9 (3.5)	68.5	312						
Sinn (2012) [48]	Australia	DHA	74.2 (7.0)	28	16	6	1950/1830	ILFC	EF	MCI at baseline	Unclear
		EPA	74.9 (5.1)	18	13						
		Placebo	73.0 (4.0)	53	15						
Tokuda (2020) [42]	Japan	DHA, EPA	67.8 (0.8)	66.7	27	6	400	WMS-verbal delayed, VF, TMT-A	MEM, EF, ATT	CN at baseline	Low
		Placebo	67.7 (1.1)	52.38	21						
Valls-Pedret (2015) [43]	Spain	ALA	66.7 (5.3)	48.2	112	36	402.225	MMSE, RAVLT, VF, DSF	GLO, MEM, EF, ATT	Constituents were determined by FoodData Central^**^ CN at baseline	Unclear
		Placebo	67.9 (5.4)	52.8	127						
van de Rest (2008) [60]	Netherlands	DHA, EPA	69.9 (3.4)	45	96	3.25, 6.5	400, 1800	Corresponding *Z* scores (see Table S2)	MEM, EF, ATT		Low
		Placebo	70.1 (3.7)	44	103						
Yurko-Mauro (2010) [44]	USA	DHA	70 (9.3)	23.14	219	6	300	MMSE, VRM, SOC	GLO, MEM, EF	CN at baseline	Unclear
		Placebo	70 (8.7)	24.69	218						

*ATT* attention, *ALA* alpha-linolenic acid, *BCAT* Basic Cognitive Aptitude Test, *CN* cognitively normal, *CNT* Category Naming Test, *CPF-z Z* score of Cogstate psychomotor function, *CVLT* California Verbal Learning Test, *DHA* docosahexaenoic acid, *DSB* Digit Span Backward, *DSF* Digit Span Forward, *DSST* Digit Symbol Substitution Test, *EF* executive function, *EPA* eicosapentaenoic acid, *FAB* frontal assessment battery, *FCSRT* Free and Cued Selective Reminding Test, *GLO* global cognition, *GCF* Global Cognition Function, *ILFC* Initial Letter Fluency Change, *LM* lexical fluency, *MAE* Mental Arithmetic Efficiency, *MCI* mild cognitive impairment, *MEM* episodic memory, *MMSE* Mini-Mental State Examination, *MoCA* Montreal Cognitive Assessment, *PCT* Perceptual Speed, *POBAV* Pojmenování OBrázků A jejich Vybavení (Picture naming and their recall), *PS* Processing Speed, *RAVLT* Rey Auditory Verbal Learning Test, *RBANS* Repeatable Battery for the Assessment of Neuropsychological Status, *RDM* delayed memory of RBANS, *RM* recognition memory, *RVC* visuospatial/constructional of RBANS, *SDMT* Symbol Digit Modalities Test, *SF* semantic fluency, *SIE* Space Imagery Efficiency, *SLM* Symbol Letter Modality, *SOC* Stockings of Cambridge, *SRT* simple reaction time, *SWM* spatial working memory, *RTot* total score of RBANS, *TICS* Telephone Interview for Cognitive Status, *TMT-A* Trail Making Test-A, *TMT-B* Trail Making Test-B, *VF* verbal fluency, *VRM* Verbal Recognition Memory, *WAIS-PRI* Wechsler Adult Intelligence Scale-perceptual reasoning index, *WAIS-PSI* Wechsler Adult Intelligence Scale-processing speed index, *WMS* Wechsler memory scale, *WMSR-AC* Wechsler memory scale revised-attention/concentration, *WMSR-VM* Wechsler memory scale revised-verbal memory

^*^median (min, max); ^**^From U.S. Department of Agriculture (https://fdc.nal.usda.gov/)

Cognitive measures employed in the trials are presented in Additional file 1: Table S2. The most frequently employed measurement was the Mini-Mental State Examination (MMSE) for global cognition [38, 39, 43, 44, 46, 49, 53, 56], the delayed recall test from Rey Auditory Verbal Learning Test (RAVLT) for episodic memory [43, 46, 50, 58], verbal fluency test for executive function [42, 43, 50–53, 60], digit symbol substitution test for processing speed [39, 46, 53], trail making test A (TMT-A) for attention [36, 42, 50, 59, 60] and block design test for visuospatial function [39, 50].

### Methodological quality

In terms of blindness and/or allocation concealment, two studies were at high risk of bias. Sala-Vila et al. acknowledged that the participants were not blinded because the intervention group was given a whole food while the placebo group was told to abstain from walnuts [50]. Hashimoto et al. also pointed out that the participants might be able to distinguish the type of intervention by odor or taste [37]. Other studies did not have a high risk of bias regarding key domains (Table 1). Supporting evidence for judging the RoB of individual trials is presented in Additional file 1: Table S3.

### Efficacy of n-3 PUFA

**Fig. 2:**
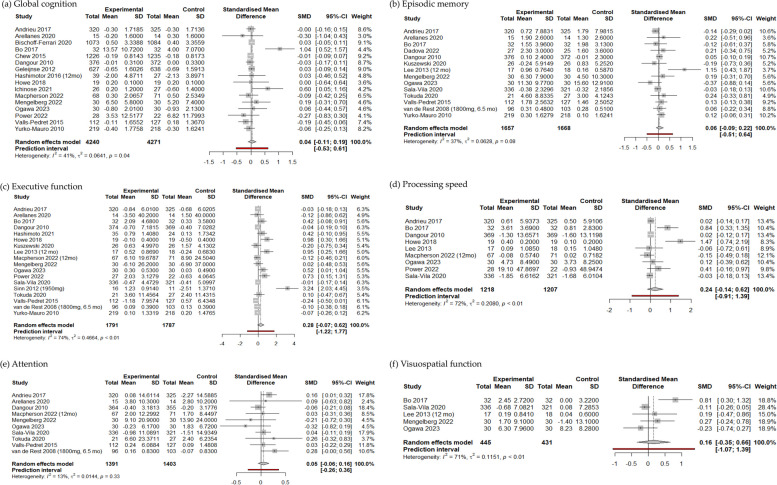
Forest plots concerning the effect of n-3 polyunsaturated fatty acids on six cognitive domains using randomized controlled trials

In the random-effects models, the intake of n-3 PUFA was not associated with the changes in global cognition (SMD [95% CI] = 0.0411 [− 0.1078, 0.1899]) and five other cognitive domains (Fig. 2). Statistical heterogeneity among the trials was substantial for the executive function (*I*^2^ = 74%, *τ*^2^ = 0.466), processing speed (*I*^2^ = 72%, *τ*^2^ = 0.208), and visuospatial function (*I*^2^ = 71%, *τ*^2^ = 0.115) [29].

**Fig. 3:**
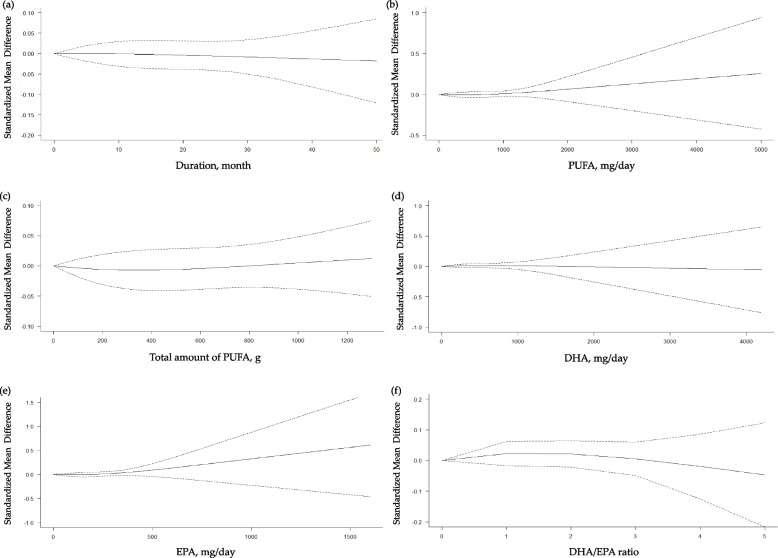
Dose–response meta-analyses for the association between n-3 polyunsaturated fatty acids (PUFA) and the global cognition. **a** Duration of intervention; **b** daily intake of n-3 PUFA; **c** total amount of n-3 PUFA taken during the study period; **d** daily intake of docosahexaenoic acid (DHA); **e** daily intake of eicosapentaenoic acid (EPA); **f** ratio of DHA to EPA taken

**Fig. 4:**
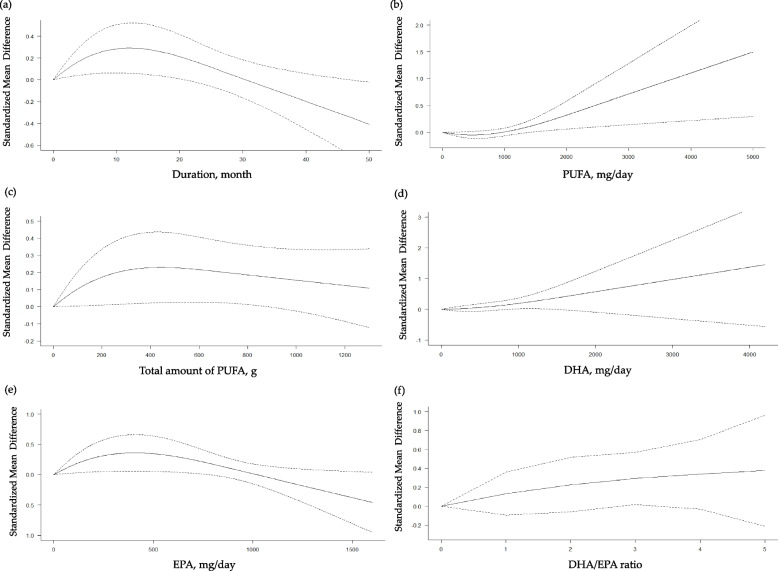
Dose–response meta-analyses for the association between n-3 polyunsaturated fatty acids (PUFA) and the executive function. **a** Duration of intervention; **b** daily intake of n-3 PUFA; **c** total amount of n-3 PUFA taken during the study period; **d** daily intake of docosahexaenoic acid (DHA); **e** daily intake of eicosapentaenoic acid (EPA); **f** ratio of DHA to EPA taken

In the subsequent restricted cubic splines models, we did not find significant non-linear dose–response relationships in terms of the duration of intervention, daily and total dose of n-3 PUFA, daily dose of DHA or EPA and the ratio of DHA to EPA with the global cognition (Fig. 3), episodic memory (Additional file 1: Fig. S1), processing speed (Additional file 1: Fig. S2), attention (Additional file 1: Fig. S3), and visuospatial function (Additional file 1: Fig. S4). On the other hand, as shown in Fig. 4a, there was a increasing beneficial effect of n-3 PUFA on the executive function up to 12 months of intervention (coefficient [95% CI] = 0.0449 [0.0101, 0.0796], *p*_*coefficient*_ = 0.0114) with a significant negative curve afterwards (coefficient [95% CI] = − 0.1896 [− 0.3326, − 0.0465], *p*_*coefficient*_ = 0.0094; goodness of fit: *χ*^2^_nonlinear_ = 6.941 versus *χ*^2^_linear_ = 4.726; *p*_nonlinearity_ = 0.031). We also found a significantly positive curve for the executive function after 500 mg/d of PUFA intake (coefficient [95% CI] = 0.0013 [0.0002, 0.0025], *p*_*coefficient*_ = 0.0249; goodness of fit: *χ*^2^_nonlinear_ = 5.715 versus *χ*^2^_linear_ = 3.492; *p*_nonlinearity_ = 0.057) (Fig. 4b). In addition, there was an increasing beneficial effect on the executive function up to 420 mg/d of EPA (coefficient [95% CI] = 0.0017 [0.0003, 0.0031], *p*_*coefficient*_ = 0.0196) with a negative curve thereafter (coefficient [95% CI] = − 0.0077 [− 0.0143, − 0.0012], *p*_*coefficient*_ = 0.0209; goodness of fit: *χ*^2^_nonlinear_ = 5.822 versus *χ*^2^_linear_ = 3.819; *p*_nonlinearity_ = 0.054) (Fig. 4e). Based on these findings, we determined to conduct subgroup analyses on the global cognition, our primary outcome, and the executive function which demonstrated significant nonlinear relationships.

### Subgroup analysis on the efficacy of n-3 PUFA by the populational blood level of DHA and EPA

In the countries where the blood level of DHA and EPA was very low, the beneficial effects of n-3 PUFA were not significant for the global cognition (Additional file 1: Fig. S5) and executive function (Additional file 1: Fig. S6).

**Fig. 5:**
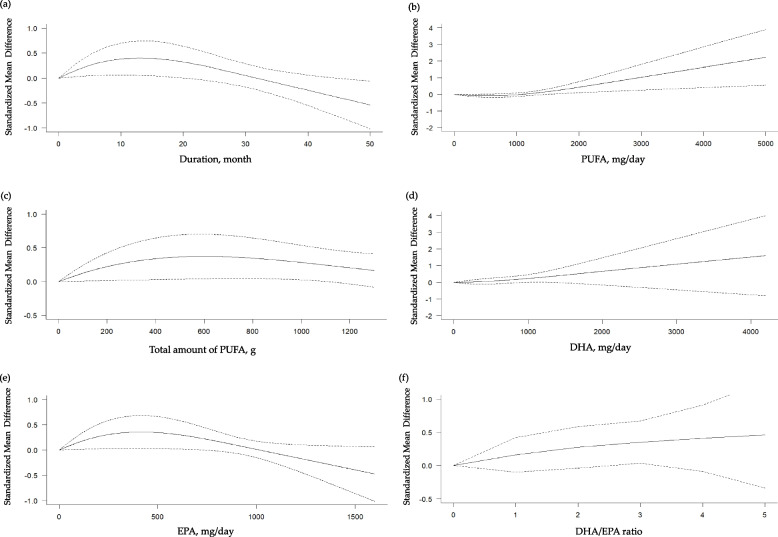
Dose–response meta-analyses for the association between n-3 polyunsaturated fatty acids (PUFA) and the executive function based on the studies from countries where the blood level of docosahexaenoic acid (DHA) + eicosapentaenoic acid (EPA) is not very low. **a** Duration of intervention; **b** daily intake of n-3 PUFA; **c** total amount of n-3 PUFA taken during the study period; **d** daily intake of DHA; **e** daily intake of EPA; **f** ratio of DHA to EPA taken

However, in case of the countries where the blood level of DHA and EPA was not very low with > 4% in erythrocyte equivalents, an increasing beneficial effect on the executive function was observed up to 12 months of intervention (coefficient [95% CI] = 0.0621 [0.0138, 0.1105], *p*_*coefficient*_ = 0.0117) followed by a descending curve (coefficient [95% CI] = − 0.2918 [− 0.5123, − 0.0713], *p*_*coefficient*_ = 0.0095; goodness of fit: *χ*^2^_nonlinear_ = 7.278 versus *χ*^2^_linear_ = 4.950; *p*_nonlinearity_ = 0.026;) (Fig. 5a). A significant ascending curve was also found for the executive function after 500 mg/d of PUFA intake (coefficient [95% CI] = 0.0016 [0.0003, 0.0029], *p*_*coefficient*_ = 0.0158; goodness of fit: *χ*^2^_nonlinear_ = 6.854 versus *χ*^2^_linear_ = 3.824; *p*_nonlinearity_ = 0.033) (Fig. 5b). An incremental beneficial effect on the executive function was also observed up to 420 mg/d of EPA (coefficient [95% CI] = 0.0016 [0.0001, 0.0031], *p*_*coefficient*_ = 0.0323) with a negative curve thereafter (coefficient [95% CI] = − 0.0071 [− 0.0138, − 0.0005], *p*_*coefficient*_ = 0.0340; goodness of fit: *χ*^2^_nonlinear_ = 4.584 versus *χ*^2^_linear_ = 3.345; *p*_nonlinearity_ = 0.101) (Fig. 5e). In regions where the level of n-3 PUFA is not very low, no substantial non-linear correlation was observed between the use of n-3 PUFA and global cognition (Additional file 1: Fig. S7).

### Subgroup analysis on the efficacy of n-3 PUFA by the presence of MCI

As for trials composed only of people with MCI, we identified two studies [36, 47] that examined the global cognition, thus preventing us to conduct any meaningful meta-analysis. For those studies that analyzed executive function, we encountered an absence of statistically significant effects attributable to n-3 PUFA (Additional file 1: Fig. S8).

In the people with normal cognition, the beneficial effects of n-3 PUFA on global cognition and the executive function were not different by the duration of intervention, daily and total doses of n-3 PUFA, and daily doses of DHA and EPA (Additional file 1: Fig. S9 and Additional file 1: Fig. S10).

### Sensitivity analyses and publication bias

When we eliminated one study at a time in a stepwise fashion, we did not observe any significant effect of an individual study on the overall estimates. However, we found that several trials have an influence on the statistical heterogeneity of the overall studies. Exclusion of the study by Bo et al. [45] led to a decrease in statistical heterogeneity concerning both global cognition and visuospatial function (Additional file 1: Table S4). Elimination of the studies by Lee et al. [46], Sinn et al. [48], and Howe et al. [57] correspondingly resulted in the reduction of statistical heterogeneity associated with episodic memory, executive function, and processing speed, respectively (Additional file 1: Table S4).

Egger’s test demonstrated that the degree of the funnel asymmetry was not significant in five random-effects models (between n-3 PUFA and the global cognition, *p* = 0.467; episodic memory, *p* = 0.226; executive function, *p* = 0.078; processing speed, *p* = 0.694; attention, *p* = 0.841) (Additional file 1: Fig. S11).

## Discussion

To the best of our knowledge, this is the first dose–response meta-analysis examining the link between n-3 PUFA intake and cognitive function in non-demented individuals exclusively of middle age or older. This study found that the beneficial effect on executive function demonstrates an upward trend within the initial 12 months of intervention. This effect is prominently observed with a daily intake surpassing 500 mg of n-3 PUFA and up to 420 mg of EPA. We also identified a descending curve following 12 months of n-3 PUFA intervention, and when the dosage of EPA exceeded 420 mg/d. Furthermore, these trends exhibit heightened significance in regions where the levels of blood DHA + EPA are not very low.

Our finding regarding the beneficial effect on the executive function is in line with a recent dose–response meta-analysis [22] and previous cross-sectional studies that suggested higher fasting plasma DHA + EPA levels [61] were associated with better executive function in dementia-free elderly individuals. The cognitive advantages attributed to n-3 polyunsaturated fatty acids (PUFA) are believed to be mediated by their influence on synaptic plasticity and neurogenesis in brain regions susceptible to oxidative stress [62]. Intriguingly, in the healthy adult population, it has been posited that vulnerability to oxidation follows a caudal-cranial gradient, with the highest vulnerability in the frontal cortex and the lowest in the spinal cord [63]. Given that executive function is primarily associated with the frontal cortex, it could elucidate why supplementation with n-3 PUFA exclusively benefits executive function. Furthermore, some scholars have suggested that the positive impact on executive function may be mediated through a reduction in the cerebrovascular lesion [64].

We identified that the beneficial effect on the executive function was not apparent for the individuals from the countries where the blood DHA + EPA level was reportedly very low with ≤ 4% in erythrocytes equivalents. It has long been suggested that this blood level is well correspond to the dietary intakes of DHA + EPA [65], and showed a similar distribution to the data obtained by nutrition surveys [66]. Researchers contend that cognitive processes cannot be singularly attributed to the accumulation of n-3 PUFA in the neural membrane; rather, the activation of various genes by dietary n-3 PUFA and their resultant products may also play a crucial role in facilitating its beneficial effects [67]. These phenomena may take place as early as conception to perinatal period characterized by an inherently gradual progression [25]. Therefore, it is tempting to propose that a habitual, long-term exposure to the n-3 PUFA might be a prerequisite to expect any beneficial effect from a high dose intake of n-3 PUFA though, from these analyses, we were unable to confirm the isolated effect of life-long intake of n-3 PUFA on the cognitive function.

Our analyses also indicated that detrimental effects to the executive function might be possible when taking n-3 PUFA for longer than 12 months or EPA for more than 420 mg/d, respectively. Several previous studies indeed suggested that a high n-3 PUFA intake might actually be associated with a low-density lipoprotein (LDL)-cholesterol-raising effect [68, 69] or a decline in the platelet count [70, 71], though their evidence is controversial [72]. Further studies on the tolerable upper limit of daily intake of n-3 PUFA, especially EPA, are needed to clarify this issue.

The current recommended guidelines for adequate n-3 PUFA intake among the adult or elderly population propose a daily intake of DHA + EPA at levels specified as follows: 250 mg according to the European Food Safety Authority [73] and Poland [74], 450 mg in the Netherlands [75], 500 mg in France [76] and Switzerland [77], between 250 and 500 mg in Belgium [78], and between 250 and 2000 mg according to the Food and Agriculture Organization of the United Nations/World Health Organization [79]. Our investigation reveals that a majority of these guidelines employ certain criteria to formulate specific recommendations, primarily focusing on the mitigation of cardiovascular diseases or the prevention of clinical deficiencies. Should subsequent research validate our findings, we posit that our results could enhance existing recommendations by incorporating cognitive perspectives. Notably, a daily n-3 PUFA intake exceeding 500 mg may warrant consideration in the refinement of these guidelines.

There are several limitations that warrant comments. Firstly, multiple cognitive tests were utilized to represent a single cognitive domain. However, efforts were made to reduce this heterogeneity by prioritizing the most commonly used cognitive test for analysis. Conversely, certain cognitive tests were found to lack specificity for the designated cognitive domain. Nonetheless, the overall results demonstrated robustness even after reassigning the test to an alternative domain (data not shown). Secondly, in several instances, the intervention material of trials claiming to be focused on n-3 PUFA did not exclusively consist of it. Minor amounts of other components such as vitamins, protein, or minerals were also mixed in. To ensure the generalizability of our analyses, we opted to exclude only those studies explicitly stating the inclusion of B vitamins in the intervention material but not in the placebo, as these were reported to be linked with cognitive benefits in certain studies [80]. Thirdly, we observed that four studies [45, 46, 48, 57] have significantly contributed to the overall statistical heterogeneity. We postulated that the utilization of distinctive cognitive assessments, such as the Basic Cognitive Aptitude Test, Initial Letter Fluency Change, or *Z* scores, might have exacerbated the heterogeneity, though sensitivity analyses for each of these studies showed robustness.

## Conclusions

In conclusion, supplementation of n-3 PUFA may offer potential advantages for executive function in the middle-aged and elderly population, particularly in individuals whose dietary DHA + EPA level is not substantially diminished.

### Supplementary Information

**Additional file 1: Table S1.** Search strategies by data sources. **Table S2.** Cognitive tests employed in each trial by their corresponding cognitive domain. **Table S3.** Detailed description of the risk of bias (RoB) for the individual studies. **Table S4.** Sensitivity analyses for each of the six cognitive domains. **Fig. S1.** Dose-response meta-analyses for the association between n-3 polyunsaturated fatty acids (PUFA) and the episodic memory. (a) duration of intervention; (b) daily intake of n-3 PUFA; (c) total amount of n-3 PUFA taken during the study period; (d) daily intake of docosahexaenoic acid (DHA); (e) daily intake of eicosapentaenoic acid (EPA); (f) ratio of DHA to EPA taken. **Fig. S2.** Dose-response meta-analyses for the association between n-3 polyunsaturated fatty acids (PUFA) and the processing speed. (a) duration of intervention; (b) daily intake of n-3 PUFA; (c) total amount of n-3 PUFA taken during the study period; (d) daily intake of docosahexaenoic acid (DHA); (e) daily intake of eicosapentaenoic acid (EPA); (f) ratio of DHA to EPA taken. **Fig. S3.** Dose-response meta-analyses for the association between n-3 polyunsaturated fatty acids (PUFA) and the attention. (a) duration of intervention; (b) daily intake of n-3 PUFA; (c) total amount of n-3 PUFA taken during the study period; (d) daily intake of docosahexaenoic acid (DHA); (e) daily intake of eicosapentaenoic acid (EPA); (f) ratio of DHA to EPA taken. **Fig. S4.** Dose-response meta-analyses for the association between n-3 polyunsaturated fatty acids (PUFA) and the visuospatial function. (a) duration of intervention; (b) daily intake of n-3 PUFA; (c) total amount of n-3 PUFA taken during the study period; (d) daily intake of docosahexaenoic acid (DHA); (e) daily intake of eicosapentaenoic acid (EPA); (f) ratio of DHA to EPA taken. **Fig. S5.** Dose-response meta-analyses for the association between n-3 polyunsaturated fatty acids (PUFA) and the global cognition based on the studies from countries where the blood level of DHA + EPA is very low. (a) duration of intervention; (b) daily intake of n-3 PUFA; (c) total amount of n-3 PUFA taken during the study period; (d) daily intake of docosahexaenoic acid (DHA); (e) daily intake of eicosapentaenoic acid (EPA); (f) ratio of DHA to EPA taken. **Fig. S6.** Dose-response meta-analyses for the association between n-3 polyunsaturated fatty acids (PUFA) and the executive function based on the studies from countries where the blood level of DHA + EPA is very low. (a) duration of intervention; (b) daily intake of n-3 PUFA; (c) total amount of n-3 PUFA taken during the study period; (d) daily intake of docosahexaenoic acid (DHA); (e) daily intake of eicosapentaenoic acid (EPA); (f) ratio of DHA to EPA taken. **Fig. S7.** Dose-response meta-analyses for the association between n-3 polyunsaturated fatty acids (PUFA) and the global cognition based on the studies from countries where the blood level of DHA + EPA is not very low. (a) duration of intervention; (b) daily intake of n-3 PUFA; (c) total amount of n-3 PUFA taken during the study period; (d) daily intake of docosahexaenoic acid (DHA); (e) daily intake of eicosapentaenoic acid (EPA); (f) ratio of DHA to EPA taken. **Fig. S8.** Dose-response meta-analyses for the association between n-3 polyunsaturated fatty acids (PUFA) and the executive function of the people with mild cognitive impairment. (a) daily intake of n-3 PUFA; (b) total amount of n-3 PUFA taken during the study period; (c) daily intake of docosahexaenoic acid (DHA); (d) daily intake of eicosapentaenoic acid (EPA); (e) ratio of DHA to EPA taken. **Fig. S9.** Dose-response meta-analyses for the association between n-3 polyunsaturated fatty acids (PUFA) and the global cognition of the cognitively normal individuals. (a) duration of intervention; (b) daily intake of n-3 PUFA; (c) total amount of n-3 PUFA taken during the study period; (d) daily intake of docosahexaenoic acid (DHA); (e) daily intake of eicosapentaenoic acid (EPA); (f) ratio of DHA to EPA taken. **Fig. S10.** Dose-response meta-analyses for the association between n-3 polyunsaturated fatty acids (PUFA) and the executive function of the cognitively normal individuals. (a) duration of intervention; (b) daily intake of n-3 PUFA; (c) total amount of n-3 PUFA taken during the study period; (d) daily intake of docosahexaenoic acid (DHA); (e) daily intake of eicosapentaenoic acid (EPA); (f) ratio of DHA to EPA taken. **Fig. S11.** Funnel plots of meta-analyses between n-3 polyunsaturated fatty acids and the (a) global cognition, (b) episodic memory, (c) executive function, (d) processing speed, (e) attention, and (f) visuospatial function.

## Data Availability

The datasets utilized and/or analyzed throughout the present study are obtainable from the corresponding author upon reasonable request.

## Abbreviations

ALA: Alpha linolenic acid
BDNF: Brain-derived neurotrophic factor
CI: Confidence interval
DHA: Docosa hexaenoic acid
EPA: Eicosa pentaenoic acid
LDL: Low-density lipoprotein
MCI: Mild cognitive impairment
MMSE: Mini-Mental Status Examination
N-3 PUFA: Omega 3 polyunsaturated fatty acids
RAVLT: Rey Auditory Verbal Learning Test
RoB: Risk of bias
SD: Standard deviation
SMD: Standardized mean difference
TMT-A: Trail making test A
