# Supplementary material for: The influence of n-3 polyunsaturated fatty acids on cognitive function in individuals without dementia: a systematic review and dose–response meta-analysis
Source: BMC Med. 2024 Mar 12;22:109. doi: 10.1186/s12916-024-03296-0 (PMC10929146; doi:10.1186/s12916-024-03296-0)
Supplement: Supplementary file 1 — Additional file 1: Table S1. Search strategies by data sources. Table S2. Cognitive tests employed in each trial by their corresponding cognitive domain. Table S3. Detailed description of the risk of bias (RoB) for the individual studies. Table S4. Sensitivity analyses for each of the six cognitive domains. Fig. S1. Dose-response meta-analyses for the association between n-3 polyunsaturated fatty acids (PUFA) and the episodic memory. (a) duration of intervention; (b) daily intake of n-3 PUFA; (c) total amount of n-3 PUFA taken during the study period; (d) daily intake of docosahexaenoic acid (DHA); (e) daily intake of eicosapentaenoic acid (EPA); (f) ratio of DHA to EPA taken. Fig. S2. Dose-response meta-analyses for the association between n-3 polyunsaturated fatty acids (PUFA) and the processing speed. (a) duration of intervention; (b) daily intake of n-3 PUFA; (c) total amount of n-3 PUFA taken during the study period; (d) daily intake of docosahexaenoic acid (DHA); (e) daily intake of eicosapentaenoic acid (EPA); (f) ratio of DHA to EPA taken. Fig. S3. Dose-response meta-analyses for the association between n-3 polyunsaturated fatty acids (PUFA) and the attention. (a) duration of intervention; (b) daily intake of n-3 PUFA; (c) total amount of n-3 PUFA taken during the study period; (d) daily intake of docosahexaenoic acid (DHA); (e) daily intake of eicosapentaenoic acid (EPA); (f) ratio of DHA to EPA taken. Fig. S4. Dose-response meta-analyses for the association between n-3 polyunsaturated fatty acids (PUFA) and the visuospatial function. (a) duration of intervention; (b) daily intake of n-3 PUFA; (c) total amount of n-3 PUFA taken during the study period; (d) daily intake of docosahexaenoic acid (DHA); (e) daily intake of eicosapentaenoic acid (EPA); (f) ratio of DHA to EPA taken. Fig. S5. Dose-response meta-analyses for the association between n-3 polyunsaturated fatty acids (PUFA) and the global cognition based on the studies from countries where the blood l [file 12916_2024_3296_MOESM1_ESM.zip › Additional file 1R5_240115.docx]

Table S1. Search strategies by data sources

| Data Sources | Search Strategy |  |  |
| --- | --- | --- | --- |
| MEDLINE (R) and Epub ahead of print, In-process & other non-indexed citations, Daily and versions (R) (Ovid SP), 1946 to Sep 2023  (Hits retrieved: 598) | 1. exp Fatty Acids, Unsaturated/ | 2. exp Fatty Acids, Omega-3/ | 3. fatty acid*.ti,ab. |
|  | 4. "polyunsaturated fat*".ti,ab. | 5. "omega-3 fatty acid*".ti,ab. | 6. "n-3 fatty acid*".ti,ab. |
|  | 7. exp Docosahexaenoic Acids/ | 8. docosahexaen*.ti,ab. | 9. DHA.ti,ab. |
|  | 10. exp Eicosapentaenoic Acid/ | 11. eicosapentaen*.ti,ab. | 12. icosapentaen*.ti,ab. |
|  | 13. EPA.ti,ab. | 14. exp alpha-Linolenic Acid/ | 15. "linolenic acid*".ti,ab. |
|  | 16. ALA.ti,ab. | 17. exp Fish Oils/ | 18. Linseed Oil/ |
|  | 19. linolenic acids/ or alpha-linolenic acid/ | 20. Flax/ | 21. (fish adj3 (diet* or nutrit* or oil* or supplement*)).ti,ab. |
|  | 22. (oil* adj3 (cod* or marin*)).ti,ab. | 23. (omega-3 or omega3 or (omega* adj5 fat*)).ti,ab. | 24. (oil* adj3 (flax* or rapeseed* or canola*)).ti,ab. |
|  | 25. (Linolen* or alpha-linolen* or alphalinolen*).ti,ab. | 26. (perilla* or linseed* or maxepa*).ti,ab. | 27. (oil* adj3 (rape or colza)).ti,ab. |
|  | 28. (marin* adj3 lipid*).ti,ab. | 29. (naudicelle* or herring* or sild).ti,ab. | 30. (clupe* adj3 hareng*).ti,ab. |
|  | 31. (whitebait or sardine* or sardina* or pilchard* or sprat* or brisling*).ti,ab. | 32. (salmo* adj3 trut*).ti,ab. | 33. (trout or bloater or kipper* or salmon or mackerel* or scomb* or conger* or tuna or tunny or tunafish or tuna-fish).ti,ab. |
|  | 34. (thunnus* or swordfish* or xiphias* or dogfish or scyliorhinus* or laks or lax).ti,ab. | 35. (crab or crabs or cancer pagurus).ti,ab. | 36. exp salmoniformes/ or tuna/ |
|  | 37. (fish adj3 capsul*).ti,ab. | 38. docosapentaen*.ti,ab. | 39. (oil* adj3 (purslane or mustard* or candlenut* or stillingia or walnut*)).ti,ab. |
|  | 40. or/1-39 | 41. *aging/ | 42. aged/ |
|  | 43. "Aged, 80 and over"/ |  | 44. middle aged/ |
|  | 45. age factors/ | 46. "old* adults".ti,ab. | 47. elderly.ti,ab. |
|  | 48. "old* age*".ti,ab. | 49. "middle age*".ti,ab. | 50. seniors.ti,ab. |
|  | 51. "senior citizens".ti,ab. | 52. pensioners.ti,ab. | 53. "aged sample".ti,ab. |
|  | 54. "aged population".ti,ab. | 55. "mild cognitive impairment".ti,ab. | 56. Mild Cognitive Impairment/ |
|  | 57. MCI.ti,ab. | 58. AAMI.ti,ab. | 59. "age-associated memory impairment".ti,ab. |
|  | 60. AACD.ti,ab. | 61. "age-associated cognitive decline".ti,ab. | 62. ACMI.ti,ab. |
|  | 63. "age-consistent memory impairment".ti,ab. | 64. ARCD.ti,ab. | 65. "age-related Cognitive Decline".ti,ab. |
|  | 66. CIND.ti,ab. | 67. "cognitive impairment no dementia".ti,ab. | 68. or/41-67 |
|  | 69. 40 and 68 | 70. *cognition/ | 71. *cognition disorders/ |
|  | 72. memory/ | 73. memory disorders/ | 74. (cognit* adj3 (func* or declin* or reduc* or impair* or improve* or deficit* or progress* or perform* or abilit*)).ti,ab. |
|  | 75. "mental perform*".ti,ab. | 76. memory.ti,ab. | 77. "episodic memory".ti,ab. |
|  | 78. Memory, Episodic/ | 79. "executive function*".ti,ab. | 80. Executive Function/ |
|  | 81. Attention/ | 82. (speed adj2 processing).ti,ab. | 83. visuospatial.ti,ab. |
|  | 84. language.ti,ab. | 85. or/70-84 | 86. 69 and 85 |
|  | 87. randomized controlled trial.pt. | 88. controlled clinical trial.pt. | 89. randomized.ab. |
|  | 90. placebo.ab. | 91. drug therapy.fs. | 92. randomly.ab. |
|  | 93. trial.ab. | 94. groups.ab. | 95. or/87-94 |
|  | 96. exp Animals/ not humans.sh. | 97. 95 not 96 | 98. 86 and 97 |
|  |  |  |  |
| EMBASE,  1974 to Sep 2023  (Hits retrieved: 546) | 1. 'polyunsaturated fatty acid'/exp | 2. ‘polyunsaturated fatty acid*’:ab,ti | 3. ‘unsaturated fatty acid’/exp |
|  | 4. 'unsaturated fatty acid ':ab,ti | 5. ‘long chain fatty acid’/exp | 6. 'long chain fatty acid*':ab,ti |
|  | 7. ‘docosahexaenoic acid’/exp | 8. docosahexaen*:ab,ti | 9. docosapentaen*:ab,ti |
|  | 10. ‘icosapentaenoic acid’/exp | 11. eicosapentaen*:ti,ab or icosapentaen*:ti,ab | 12. ‘linolenic acid’/exp |
|  | 13. Linolen*:ab,ti or alpha-linolen*:ab,ti or alphalinolen*:ab,ti | 14. 'alpha-linolenic acid':ab,ti | 15. salmoniformes/de or tuna/de |
|  | 16. ‘fish oil’/de | 17. ‘linseed oil’/de | 18. Flax/de |
|  | 19. ‘omega 3 fatty acid’/de | 20. (fish near/3 diet*):ab,ti OR (fish near/3 nutrit*):ab,ti OR (fish near/3 oil*):ab,ti OR (fish near/3 supplement*):ab,ti | 21. (oil* near/3 cod*):ab,ti OR (oil* near/3 marin*):ab,ti |
|  | 22. omega-3:ab,ti OR omega3:ab,ti OR (omega* near/5 fat*):ab,ti | 23. (oil* near/3 flax*):ab,ti OR (oil* near/3 rapeseed*):ab,ti OR (oil* near/3 canola*):ab,ti | 24. perilla*:ab,ti OR linseed*:ab,ti OR maxepa*:ab,ti |
|  | 25. (marin* near/3 lipid*):ab,ti | 26. (naudicelle* or herring* or sild):ab,ti | 27. (clupe* near/3 hareng*):ab,ti |
|  | 28. (whitebait or sardine* or sardina* or pilchard* or sprat* or brisling*):ab,ti | 29. (salmo* near/3 trut*):ab,ti | 30. (trout or bloater or kipper* or salmon or mackerel* or scomb* or conger* or tuna or tunny or tunafish or tuna-fish):ab,ti |
|  | 31. (thunnus* or swordfish* or xiphias* or dogfish or scyliorrhinus* or laks or lax):ab,ti | 32. crab:ab,ti OR crabs:ab,ti OR (cancer near/3 pagarus):ab,ti | 33. salmonine/exp |
|  | 34. (fish near/3 capsul*):ab,ti | 35. ALA:ab,ti or DHA:ab,ti or DPA:ab,ti or EPA:ab,ti | 36. #1 OR #2 OR #3 OR #4 OR #5 OR #6 OR #7 OR #8 OR #9 OR #10 OR #11 OR #12 OR #13 OR #14 OR #15 OR #16 OR #17 OR #18 OR #19 OR #20 OR #21 OR #22 OR #23 OR #24 OR #25 OR #26 OR #27 OR #28 OR #29 OR #30 OR #31 OR #32 OR #33 OR #34 OR #35 |
|  | 37. aging/de | 38. aged/de | 39. 'middle aged'/de |
|  | 40. 'mild cognitive impairment'/de | 41. 'mild cognitive impairment':ab,ti | 42. MCI:ab,ti |
|  | 43. AAMI:ab,ti | 44. 'age-associated memory impairment':ab,ti | 45. AACD:ab,ti |
|  | 46. 'age-associated cognitive decline':ab,ti | 47. ACMI:ab,ti | 48.'age-consistent memory impairment':ab,ti |
|  | 49. ARCD:ab,ti | 50.'age-related cognitive decline':ab,ti | 51. CIND:ab,ti |
|  | 52. 'cognitive impairment no dementia':ab,ti | 53.'middle age*':ab,ti | 54. 'old* age*':ab,ti |
|  | 55. 'old* adults':ab,ti | 56. 'senior citizens':ab,ti | 57. seniors:ab,ti |
|  | 58. pensioners:ab,ti | 59. 'aged sample':ab,ti | 60. 'aged population':ab,ti |
|  | 61. #37 OR #38 OR #39 OR #40 OR #41 OR #42 OR #43 OR #44 OR #45 OR #46 OR #47 OR #48 OR #49 OR #50 OR #51 OR #52 OR #53 OR #54 OR #55 OR #56 OR #57 OR #58 OR #59 OR #60 | 62. ‘cognition’/exp | 63. ‘cognition disorders’/de |
|  | 64. ‘episodic memory’/de OR memory/de | 65. ‘memory disorder’/de | 66. dementia/de |
|  | 67. ‘Alzheimer disease’/de | 68. dementia*:ab,ti | 69. Alzheimer*:ab,ti |
|  | 70. cognition:ab,ti | 71. cognitive:ab,ti | 72. #62 OR #63 OR #64 OR #65 OR #66 OR #67 OR #68 OR #69 OR #70 OR #71 |
|  | 73. #36 AND #61 AND #72 | 74. ‘randomized controlled trial’/de | 75.‘controlled clinical trial’/de |
|  | 76. placebo:ab | 77. (random* NEAR/2divide*):ab,ti | 78. (random* NEAR/2allocate*):ab,ti |
|  | 79. trial:ab | 80. ‘double-blind*’:ab,ti | 81.‘single-blind*’:ab,ti |
|  | 82. #74 OR #75 OR #76 OR #77 OR #78 OR #79 OR #80 OR #81 | 83. #73 AND #82 | 84. [embase]/lim |
|  | 85.#83 AND #84 |  |  |
|  |  |  |  |
| PsycINFO (EBSCOhost),  Jan 1806 to Sep 2023  (Hits retrieved: 171) | S1. MJ “fatty acids” + | S2. MJ “salmon” + | S3. TI “unsaturated fatty acid*” OR AB “unsaturated fatty acid*” |
|  | S4. TI “omega-3 fatty acid*” OR AB “omega-3 fatty acid*” | S5. TI “polyunsaturated fat*” OR AB “polyunsaturated fat*” | S6. TI “n-3 fatty acid*” OR AB “n-3 fatty acid*” |
|  | S7. TI “docosahexaen*” OR AB “docosahexaen*” | S8. TI DHA OR AB DHA | S9. TI “eicosapentaen*” OR AB “eicosapentaen*” |
|  | S10. TI “icosapentaen*” OR AB “icosapentaen*” | S11. TI EPA OR AB EPA | S12. TI “linolenic acid*” OR AB “linolenic acid*” |
|  | S13. TI ALA OR AB ALA | S14. TI “fish oil*” OR AB “fish oil*” | S15. TI “Linseed Oil” OR AB “Linseed Oil” |
|  | S16. TI flax OR AB flax | S17. TI “fish diet*” OR AB “fish diet*” | S18. TI “cod oil*” OR AB “cod oil*” |
|  | S19. TI “omega* fat*” OR AB “omega* fat*” |  | S20. S1 OR …… OR S19 |
|  |  |  |  |
|  | S21. MJ aging + | S22. MJ "cognitive impairment" + | S23. TI “cognit* impair*” OR AB “cognit* impair*” |
|  | S24. TI MCI OR AB MCI | S25. TI "mild cognitive impairment" OR AB "mild cognitive impairment" | S26. TI AAMI OR AB AAMI |
|  | S27. TI "age-associated memory impairment" OR AB "age-associated memory impairment" | S28. TI AACD OR AB AACD | S29. TI "age-associated cognitive decline" OR AB "age-associated cognitive decline" |
|  | S30. TI ACMI OR AB ACMI | S31. TI "age-consistent memory impairment" OR AB "age-consistent memory impairment" | S32. TI ARCD OR AB ARCD |
|  | S33. TI "age-related cognitive decline" OR AB "age-related cognitive decline" | S34. TI CIND OR AB CIND | S35. TI "cognitive impairment no dementia" OR AB "cognitive impairment no dementia" |
|  | S36. TI “old* age*” OR AB “old* age*” | S37. TI elderly OR AB elderly | S38. TI “middle age*” OR AB “middle age*” |
|  | S39. TI “old* adults” OR AB “old* adults” | S40. TI seniors OR AB seniors | S41. TI “senior citizens” OR AB “senior citizens” |
|  | S42. TI pensioners OR AB pensioners | S43. MJ cognition + | S44. MJ dementia + |
|  | S45. S21 OR …… OR S44 | S46. MA "Randomized Controlled Trials" | S47. AB randomly |
|  | S48. AB placebo | S49. AB groups | S50. AB RCT |
|  | S51. TX "double blind*” | S52. TX "single blind*” | S53. TX "controlled clinical trial" |
|  | S54. TI randomised | S55. TI randomized | S56. S46 OR …… S55 |
|  | S57. S20 AND S45 AND S56 |  |  |
|  |  |  |  |
| CINAHL (EBSCOhost), 1961 to Sep 2023  (Hits retrieved: 56) | S1. MH "Fatty Acids, Unsaturated+" | S2. MH “Fatty Acids, Omega-3+” | S3. MH “Docosahexaenoic Acids+” |
|  | S4. MH “Eicosapentaenoic Acid+” | S5. MH “alpha-Linolenic Acid+” | S6. MH “Fish Oils+” |
|  | S7. MH “Linseed Oil” | S8. (MH “linolenic acids”) OR (MH “alpha-linolenic acid”) | S9. MH “Flaxseed” |
|  | S10. TI “fatty acid*” OR AB “fatty acid*” | S11. TI “polyunsaturated fat*” OR AB “polyunsaturated fat*” | S12. TI "omega-3 fatty acid*" OR AB "omega-3 fatty acid*" |
|  | S13. TI "n-3 fatty acid*" OR AB "n-3 fatty acid*" | S14. TI docosahexaen* OR AB docosahexaen* | S15. TI DHA OR AB DHA |
|  | S16. TI eicosapentaen* OR AB eicosapentaen* | S17. TI icosapentaen* OR AB icosapentaen* | S18. TI EPA OR AB EPA |
|  | S19. TI "linolenic acid*" OR AB "linolenic acid*" | S20. TI ALA OR AB ALA | S21. TI docosapentaen* OR AB docosapentaen* |
|  | S22. TI “omega* fat*” OR AB “omega* fat*” | S23. S1 OR …… OR S22 | S24. MH "Aging" |
|  | S25. (MH "Aged") OR (MH "Aged, 80 and Over") | S26. MH "Middle Age" | S27. TI MCI OR AB MCI |
|  | S28. TI AAMI OR AB AAMI | S29. TI ACMI OR AB ACMI | S30. TI ARCD OR AB ARCD |
|  | S31. TI CIND OR AB CIND | S32. TI AACD OR AB AACD | S33. TI "Mild Cognitive Impairment" OR AB "Mild Cognitive Impairment" |
|  | S34. TI "age-associated memory impairment" OR AB "age-associated memory impairment" | S35. TI "age-consistent memory impairment" OR AB "age-consistent memory impairment" | S36. TI "age-related cognitive decline" OR AB "age-related cognitive decline" |
|  | S37. TI "cognitive impairment no dementia" OR AB "cognitive impairment no dementia" | S38. TI "age-associated cognitive decline" OR AB "age-associated cognitive decline" | S39. TI elderly OR AB elderly |
|  | S40. TI "old* adults" OR AB "old* adults" | S41. TI "old* age*" OR AB "old* age*" | S42. TI pensioners OR AB pensioners |
|  | S43. TI seniors OR AB seniors | S44. TI "senior citizen*" OR AB "senior citizen*" | S45. TI "age* sample" OR AB "age* sample" |
|  | S46. TI "age* population" OR AB "age* population" | S47. S24 OR …… OR S46 | S48. (MH "Cognition") OR (MH "Cognition Disorders") OR (MH "Delirium, Dementia, Amnestic, Cognitive Disorders") |
|  | S49. TI cognition OR AB cognition | S50. TI memory OR AB memory | S51. (MH "Memory") OR (MH "Memory Disorders") OR (MH "Memory, Short Term") |
|  | S52. TI "executive function" OR AB "executive function" | S53. TI "cognitive* declin*" OR AB "cognitive* declin*" | S54. TI "cognitive* improv*" OR AB "cognitive* improv*" |
|  | S55. TI "cognitive deficit*" OR AB "cognitive deficit*" | S56. TI "mental perform*" OR AB "mental perform*" | S57. TI dementia OR AB dementia |
|  | S58. TI alzheimer* OR AB alzheimer* | S59. MH "Dementia+" | S60. S48 OR …… OR S59 |
|  | S61. MH "Randomized Controlled Trials" | S62. AB randomly | S63. AB placebo |
|  | S64. AB groups | S65. AB RCT | S66. TI "double blind*" OR AB "double blind*" |
|  | S67. TI "single blind*" OR AB "single blind*" | S68. TI "controlled clinical trial" OR AB "controlled clinical trial" | S69. TI randomised |
|  | S70. TI randomized | S71. S61 OR …… OR S70 | S72. S23 AND S47 AND S60 AND S71 |
|  |  |  |  |
| Cochrane Central Register of Controlled Trials (CENTRAL), Sep 2023  (Hits retrieved: 482) | #1 MeSH descriptor: [Fish Oils] explode all trees | #2 MeSH descriptor: [Linseed Oil] this term only | #3 MeSH descriptor: [Linolenic Acids] in all MeSH products |
|  | #4 MeSH descriptor: [Fatty Acids, Omega-3] explode all trees | #5 (fish near/3 oil*):ti,ab,kw | #6 (oil* near/3 (cod* or marin*)):ti,ab,kw |
|  | #7 (omega-3 or omega3 or (omega* near/5 fat*)):ti,ab,kw | #8 (eicosapentaen*):ti,ab,kw | #9 (docosahexaen*):ti,ab,kw |
|  | #10 (oil* near/3 (flax* or rapeseed* or canola*)):ti,ab,kw | #11 (Linolen* or alpha-linolen* or alphalinolen*):ti,ab,kw | #12 (perilla* or linseed* or maxepa*):ti,ab,kw |
|  | #13 (oil* near/3 (rape or colza)):ti,ab,kw | #14 (marin* near/3 lipid*):ti,ab,kw | #15 (naudicelle* or herring* or sild):ti,ab,kw |
|  | #16 (clupe* near/3 hareng*):ti,ab,kw | #17 (whitebait or sardine* or sardina* or pilchard* or sprat* or brisling*):ti,ab,kw | #18 (salmo* near/3 trut*):ti,ab,kw |
|  | #19 (trout or bloater or kipper* or salmon or mackerel* or scomb* or conger* or tuna or tunny or tunafish or tuna-fish):ti,ab,kw | #20 (thunnus* or swordfish* or xiphias* or dogfish or scyliorrhinus*):ti,ab,kw | #21 (crab or crabs or (cancer pagarus)):ti,ab,kw |
|  | #22 (DHA or EPA):ti,ab,kw | #23 MeSH descriptor: [Salmoniformes] explode all trees | #24 MeSH descriptor: [Tuna] this term only |
|  | #25 MeSH descriptor: [alpha-Linolenic Acid] this term only | #26 MeSH descriptor: [Flax] this term only | #27 (fish near/3 (diet* or capsul* or nutrit* or supplement*)):ti,ab,kw |
|  | #28 (icosapentaen* or docosapentaen*):ti,ab,kw | #29 (oil* near/3 (purslane or mustard* or candlenut* or stillingia or walnut*)):ti,ab,kw | #30 (laks or lax):ti,ab,kw |
|  | #31 (ALA or DPA):ti,ab,kw | #32 (algal near oil*):ti,ab,kw |  |
|  |  | #33. #1 OR …… OR #32 | #34. MeSH descriptor: [Aged] in all MeSH products |
|  | #35. MeSH descriptor: [Aged, 80 and over] this term only | #36. MeSH descriptor: [Middle Aged] this term only | #37. MeSH descriptor: [Age Factors] this term only |
|  | #38. (old* adults):ti,ab,kw | #39. (elderly):ti,ab,kw | #40. (old* age*):ti,ab,kw |
|  | #41. (middle age*):ti,ab,kw | #42. (seniors):ti,ab,kw | #43. (senior citizens):ti,ab,kw |
|  | #44. (pensioners):ti,ab,kw | #45. (aged sample):ti,ab,kw | #46. (aged population):ti,ab,kw |
|  | #47. (mild cognitive impairment):ti,ab,kw | #48. MeSH descriptor: [Cognitive Dysfunction] this term only | #49. (MCI):ti,ab,kw |
|  | #50. (AAMI):ti,ab,kw | #51. (age-associated memory impairment):ti,ab,kw | #52. (AACD):ti,ab,kw |
|  | #53. (age-associated cognitive decline):ti,ab,kw | #54. (ACMI):ti,ab,kw | #55. (age-consistent memory impairment):ti,ab,kw |
|  | #56. (ARCD):ti,ab,kw | #57. (age-related Cognitive Decline):ti,ab,kw | #58. (CIND):ti,ab,kw |
|  | #59. (cognitive impairment no dementia):ti,ab,kw | #60. #34 OR …… OR #59 | #61. (cognitive impairment no dementia):ti,ab,kw |
|  | #62. MeSH descriptor: [Cognition Disorders] this term only | #63. MeSH descriptor: [Memory] this term only | #64. MeSH descriptor: [Memory Disorders] this term only |
|  | #65. (cognit* NEAR/3 (func* OR declin* OR reduc* OR impair* OR improve* OR deficit* OR progress* OR perform* OR abilit*)):ti,ab,kw | #66. (mental perform*):ti,ab,kw | #67. (memory):ti,ab,kw |
|  | #68. MeSH descriptor: [Memory, Episodic] this term only | #69. (executive function*):ti,ab,kw |  |
|  |  | #70. MeSH descriptor: [Attention] this term only | #71. (speed NEAR/2 processing):ti,ab,kw |
|  | #72. (visuospatial):ti,ab,kw | #73. (language):ti,ab,kw | #74. #61 OR …… OR #73 |
|  | #75. randomized controlled trial:pt | #76. controlled clinical trial:pt | #77. (randomized):ab |
|  | #78. (placebo):ab | #79. (randomly):ab | #80. (trial):ab |
|  | #81. (groups):ab | #82. #75 OR …… OR #81 | #83. #33 AND #60 AND #74 AND #82 |
|  |  |  |  |
| International Clinical Trials Registry Platform Search Portal  Sep 2023 (Hits retrieved: 187) | **A:** in the Condition box: cognition OR “mild cognitive impairment” OR elderly OR “aged subjects” OR “older adults” OR “middle aged”  **B:** in the Intervention box: “fatty acid*, unsaturated” OR “fatty Acid*, Omega-3” OR “fatty acid*” OR “polyunsaturated fat*” OR “omega-3 fatty acid*” OR “n-3 fatty acid*” OR “docosahexaen* acid” OR “eicosapentaen* acid” OR “icosapentaen* acid” OR “linolenic acid*” OR “diet* suppl*”  **A and B**  Recruitment status is: ALL | | |
|  |  |  |  |
|  |  |  |  |

Table S2. Cognitive tests employed in each trial by their corresponding cognitive domain.

| Cognitive domain | Cognitive tests | Studies |
| --- | --- | --- |
| Global cognitive function | Mini-Mental State Examination | Andrieu 2017; Geleijnse 2012; Hashimoto 2016; Ichinose 2021; Lee 2013, Valls-Pedret 2015; Yurko-Mauro 2010; Ogawa 2023 |
|  | Telephone Interview for Cognitive Status (TICS) | Chew 2015 |
|  | Repeatable Battery of the Assessment of Neuropsychological Status (RBANS) | Power 2020; Mengelberg 2022 |
|  | Composite Z score – total of the memory, processing speed and executive function | Andrieu 2017 |
|  | Montreal Cognitive Assessment (MoCA) | Arellanes 2020; Bischoff-Ferrari 2020; Macpherson 2022 |
|  | Basic Cognitive Aptitude Tests (BCATs) | Bo 2017 |
|  | Composite scores of AREDS2 Cognitive Battery Tests | Chew 2015 |
|  | Abbreviated Mental Test (AMT) | Mahmoudi 2014 |
|  | Z score of overall performance encompassing oral trail making test, tapping, serial subtraction of 3, high number tap, letter search, visual warning, stroop color-word test, multi-tasking task, N-back test, and go no-go test. | Howe 2018 |
| Episodic memory | Free recall task | Andrieu 2017; van de Rest 2008 |
|  |  |  |
|  | Logical Memory Part I (immediate recall) from Wechsler Memory Scale (WMS) | Chew 2015; Dangour 2010; Ogawa 2023 |
|  | Word list recall from TICS | Chew 2015; |
|  | Words recalled at delayed recall of list A from California Verbal Learning Test (CVLT) | Arellanes 2020; Dangour 2010; Mengelberg 2022 |
|  | Spatial memory, delayed recall | Dangour 2010 |
|  | Delayed recall from Rey Auditory Verbal Learning Test (RAVLT) | Kuszewski 2020; Lee 2013; Sala-Vila 2020; Valls-Pedret 2015 |
|  | Learning from Rey Auditory Verbal Learning Test (RAVLT) | Sala-Vila 2020; Valls-Pedret 2015 |
|  | Visual reproduction I from WMS | Tokuda 2020 |
|  | Visual reproduction II from WMS | Tokuda 2020 |
|  | CANTAB Paired Associate Learning (PAL) | Valls-Pedret 2015 |
|  | Picture Sequence Memory Test | Kuszewski 2020 |
|  | Rey complex figure, immediate recall | Sala-Vila 2020 |
|  | Rey complex figure, delayed recall | Tokuda 2020 |
|  | Recognition form Basic Cognitive Aptitude Tests (BCATs) | Bo 2017 |
|  | Composite Z score: (ZCVLT sum of words recalled + ZCVLT delayed recall + Zlocation memory + Zlocation memory-delayed + Zstory recall + Zstory recall-delayed)/6 | Dangour 2010 |
|  | Delayed recall of WMS-III Recall paragraph | Chew 2015 |
|  | POBAV, Pojmenování OBrázků A jejich Vybavení (Picture naming and their recall) | Dadova 2022 |
|  | Z score of visual reproduction I and II, immediate recall of RAVLT, delayed recall of RAVLT, and digit span backward | Lee 2013 |
| Executive function | Verbal fluency test (category Naming Test; Semantic Verbal Fluency Test; Animal category; Issacs set test; letter fluency; alternating fluency) | Andrieu 2017; Chew 2015; Dangour 2010; Sala-Vila 2020; Tokuda 2020; Valls-Pedret 2015; van de Rest 2008 |
|  | Trail Making Test B | Arellanes 2020; Andrieu 2017; Sala-Vila 2020; Tokuda 2020; Macpherson 2022; Mengelberg 2022 |
|  | Controlled Oral Word Association Test | Andrieu 2017 |
|  | Backward Counting task; digit span backward from WAIS; Digit span backward + forward from WAIS-R | Chew 2015; Dangour 2010; Lee 2013; Sala-Vila 2020; Tokuda 2020; Valls-Pedret 2015 |
|  | Composite Z score: (digit span backward + verbal fluency)/2 | Dangour 2010 |
|  | Stroop Test; Stroop interference; Stroop Color time; Stroop Color-Word time; Stroop congruent, response time; Stroop incongruent, response time | Sala-Vila 2020; Tokuda 2020 |
|  | CANTAB Spatial Working Memory (SWM) | Power 2022 |
|  | Mental arithmetic efficiency (MAE), working memory of Basic Cognitive Aptitude Tests (BCATs) | Bo 2017 |
|  | Keio version Wisconsin Card Sorting Test (KWCST) categories achieved (CA) | Tokuda 2020 |
|  | Color Trail Test part 1 and part 2 | Valls-Pedret 2015 |
|  | Frontal assessment battery | Hashimoto 2021 |
|  | Z score of paper trail making test | Howe 2018 |
|  | Cognitive flexibility involving dimensional card sorting test, Flanker inhibitory control and attention test, and trail making test. | Kuszewski 2020 |
|  | Z score of clock drawing test and digit span forward | Lee 2013 |
|  | Z score of Cogstate psychomotor function | Macpherson 2022 |
| Processing speed | **Simple reaction time** | Dangour 2010; |
|  | Choice reaction time | Dangour 2010; |
|  | Digit Symbol Substitution Test score from WAIS-R | Andrieu 2017; Lee 2013; Ogawa 2023 |
|  | symbol letter modality test [number correct]; Symbol Digit Modalities Test, no. correct; Symbol letter modality, n correct in 90 sec | Dangour 2010; Sala-Vila 2020 |
|  | Composite Z score: [(Zletter cancellation + Zsimple reaction time + Zsymbol-letter substitution)/4] | Dangour 2010 |
|  | Cogstate psychomotor function Z score | Macpherson 2022 |
|  | Perceptual speed of Basic Cognitive Aptitude Tests (BCATs) | Bo 2017 |
|  | Z score of reaction time | Howe 2018 |
| Attention | Trail Making Test A | Arellanes 2020; Sala-Vila 2020; Tokuda 2020; van de Rest 2008; Macpherson 2022 |
|  | letter cancellation task | Dangour 2010 |
|  | digit span forward | Dangour 2010; Sala-Vila 2020; Valls-Pedret 2015; Mengelberg 2022 |
|  | Continuous Performance Test, detectability and reaction time | Sala-Vila 2020 |
| Visuospatial function |  |  |
|  | Block design from WAIS-III | Sala-Vila 2020; Ogawa 2023 |
|  | Space imagery efficiency (SIE) of Basic Cognitive Aptitude Tests (BCATs) | Bo 2017 |
|  | Rey Complex Figure Test, copy | Sala-Vila 2020; |
|  | Visual Object and Space Perception battery | Sala-Vila 2020 |
|  | Repeatable Battery of the Assessment of Neuropsychological Status (RBANS) Visuospatial/Constructional test | Mengelberg 2022 |
|  | Z score of block design and matrix reasoning | Lee 2013 |

Table S3. Detailed description of the risk of bias (RoB) for the individual studies.

| Andrieu 2017   \| Bias \| Judge \| Support \| \| --- \| --- \| --- \| \| Random sequence generation  (selection bias) \| Low \| Quote: A computer-generated randomisation procedure (done by ClinInfo, a sub contractor) was used with block sizes of eight and stratification by centre. \| \| Allocation concealment  (selection bias) \| Low \| Quote: A clinical research assistant, who was not involved in the assessment of participants, used a centralised interactive voice response system to identify which group to allocate the participant to, and which lot number to administer. \| \| Blinding of participants and  personnel (performance bias) \| Low \| Quote: All participants and study staff were blinded to polyunsaturated fatty acid or placebo assignment—both sets of capsules looked and tasted identical. \| \| Blinding of outcome  assessment (detection bias) \| Low \| Quote: All participants and study staff were blinded to polyunsaturated fatty acid or placebo assignment—both sets of capsules looked and tasted identical.  In view of the nature of the multidomain intervention, the study was unblinded for this component, but the independent neuropsychologists who were trained to assess cognitive outcomes were blinded to group assignment.  Data analysts were not blinded to group assignment, but two data managers, one statistician (CC) and two physicians (SA and BV) did a blinded data review. \| \| Incomplete outcome data  (attrition bias) \| Low \| Quote: completion rates were similar between groups (figure).  The main reasons for early discontinuation were: participants’ decision, adverse events, death, and loss to follow-up (figure). Reasons for early discontinuation did not differ significantly between groups at 36 months \| \| Selective reporting (reporting  bias) \| Low \| Quote: The trial was registered with ClinicalTrials. gov (NCT00672685).  Because of advances in the field since our trial was designed in 2007, we decided to modify the primary outcome from one cognitive test to a composite cognitive score, which is now thought to be a better endpoint. This protocol amendment was submitted to the local ethical committee on Feb 2, 2015, and was subsequently approved. \| \| Other bias \| Low \| Comment: No other sources of bias identified \|   Arellanes 2020   \| Bias \| Judge \| Support \| \| --- \| --- \| --- \| \| Random sequence generation  (selection bias) \| Low \| Comment: No other sources of bias identified. \| \| Allocation concealment  (selection bias) \| Low \| Quote: A randomization sequence was developed by the biostatistics team, with randomization fidelity monitored by the trial data manager/analyst. Only the trial statistician and one designated research coordinator had access to the randomization list. \| \| Blinding of participants and  personnel (performance bias) \| Low \| Quote: Only the trial statistician and one designated research coordinator had access to the randomization list. \| \| Blinding of outcome  assessment (detection bias) \| Unclear \| No information \| \| Incomplete outcome data  (attrition bias) \| Low \| Comment: No other sources of bias identified. \| \| Selective reporting (reporting  bias) \| Low \| Quote: The trial was registered with clinical trials.gov (NCT02541929). \| \| Other bias \| Low \| Quote: Although this was a randomized trial, there were significantly fewer males and lower baseline fasting triglyceride levels in the DHA treatment arm \|   Bischoff-Ferrari 2020   \| Bias \| Judge \| Support \| \| --- \| --- \| --- \| \| Random sequence generation  (selection bias) \| Low \| Quote: using block randomization (block sizes of 16 individuals) stratified by recruitment center, prior fall, sex, and age (70-84 years or ≥85 years)  A central randomization center in Switzerland, supported by trial software, was responsible for the blinding \| \| Allocation concealment  (selection bias) \| Low \| Quote: A central randomization center in Switzerland, supported by trial software, was responsible for the blinding, treatment allocation, and study intervention labeling. \| \| Blinding of participants and  personnel (performance bias) \| Low \| Quote: Participants received2 gel capsules per day (vitamin D or placeboandomega-3s or placebo), identical in size, appearance, taste, and weight.  All capsules had coatings to prevent unblinding by aftertaste \| \| Blinding of outcome  assessment (detection bias) \| Low \| Quote: Participants, staff dispensing study pills and collecting outcomes, and data analysts were masked to group assignment. \| \| Incomplete outcome data  (attrition bias) \| Low \| Quote: The withdrawal rate was 11.9%, with no difference in withdrawal rates across the8 treatment groups (P = .52) \| \| Selective reporting (reporting  bias) \| Low \| Quote: TRIAL REGISTRATION ClinicalTrials.gov Identifier: NCT01745263 \| \| Other bias \| Low \| Comment: No other sources of bias identified \|   Bo 2017   \| Bias \| Judge \| Support \| \| --- \| --- \| --- \| \| Random sequence generation  (selection bias) \| Low \| Quote: The randomization sequence was computer-generated by a blinded statistician not involved in  data collection or analysis according to age and gender. \| \| Allocation concealment  (selection bias) \| Low \| Quote: The randomization sequence was computer-generated by a blinded statistician not involved in  data collection or analysis according to age and gender. \| \| Blinding of participants and  personnel (performance bias) \| Unclear \| Comment: No information \| \| Blinding of outcome  assessment (detection bias) \| Unclear \| Comment: No information \| \| Incomplete outcome data  (attrition bias) \| Low \| Quote: Another limitation was the high dropout rate (25%), which could bias the results via intention-to-treat analysis. The dropout rate was similar in both groups, and baseline characteristics of the dropouts were comparable to those participants included in the final analysis. \| \| Selective reporting (reporting  bias) \| Low \| Quote: This study is ChiCTR-TRC-14004625 in the Chinese Clinical Trial Registry (http://www.chictr.org.cn). \| \| Other bias \| Low \| Comment: No other sources of bias identified \|   Chew 2015   \| Bias \| Judge \| Support \| \| --- \| --- \| --- \| \| Random sequence generation  (selection bias) \| Low \| Quote: The primary and secondary randomizations were stratified by clinical center and AMD category (bilateral large drusen or large drusen in one eye and advanced AMD in the fellow eye) using randomly permuted blocks of varying sizes. \| \| Allocation concealment  (selection bias) \| Low \| Quote: Each treatment was assigned 5 bottle numbers. Bottle numbers were issued via an electronic randomization system for each participant once study eligibility was verified. The assigned bottle number was used to distribute the study treatment(s).  AREDS2 Coordinating center personnel involved in creating the randomization system had access to the bottle number/treatment assignments. \| \| Blinding of participants and  personnel (performance bias) \| Low \| Quote: The investigational products matched the placebos in size, shape, and taste.  Participants and study personnel were masked to treatment assignment. \| \| Blinding of outcome  assessment (detection bias) \| Low \| Quote: Participants and study personnel were masked to treatment assignment.  Investigators, also masked to all medical data and treatment assignments, conducted the analyses. \| \| Incomplete outcome data  (attrition bias) \| Low \| However, among those who did not participate, there was a relatively greater proportion assigned to lutein/zeaxanthin (eTable 2 in Supplement 2). \| \| Selective reporting (reporting  bias) \| Low \| Quote: TRIAL REGISTRATION clinicaltrials.gov Identifier: NCT00345176 \| \| Other bias \| Low \| Comment: No other sources of bias identified. \|   Dadova 2022   \| Bias \| Judge \| Support \| \| --- \| --- \| --- \| \| Random sequence generation  (selection bias) \| Unclear \| Quote: Women were randomized into 2 groups matched for age, BMI, and VO2peak (each group containing 27-28 subjects). \| \| Allocation concealment  (selection bias) \| Unclear \| Quote: Participants were randomly divided into either an exercise and Calanus supplementation group (Cal-EX) or exercise and placebo supplementation group (Pla-EX). \| \| Blinding of participants and  personnel (performance bias) \| Low \| Quote: Neither the subjects nor the investigators who came into personal contact with the subjects were informed about who was taking supplementation and who was taking placebo. \| \| Blinding of outcome  assessment (detection bias) \| Low \| Quote: Neither the subjects nor the investigators who came into personal contact with the subjects were informed about who was taking supplementation and who was taking placebo. \| \| Incomplete outcome data  (attrition bias) \| Low \| Quote: The dropout during the ET intervention (n = 2) was caused mainly by newly diagnosed health problems, which were not associated with ET itself; 2 subjects were excluded from the AT analysis because they refused AT biopsy at the end of the program. \| \| Selective reporting (reporting  bias) \| Low \| Quote: The study was registered as EXODYA (Effect of EXercise training and Omega-3 fatty acids on metabolic health and DYsfunction of Adipose tissue in elderly) research project, ClinicalTrials.gov registration no (NCT03386461). \| \| Other bias \| Low \| Comment: No other sourced of bias identified. \|   Dangour 2010   \| Bias \| Judge \| Support \| \| --- \| --- \| --- \| \| Random sequence generation  (selection bias) \| Low \| Quote: Research nurses telephoned a central computerized randomization service to obtain treatment-allocation codes previously generated by the trial statistician.  Randomization was minimized by age group (70–74 and 75–79 y) and general practice to ensure a balance across trial arms. \| \| Allocation concealment  (selection bias) \| Low \| Quote: Research nurses telephoned a central computerized randomization service to obtain treatment-allocation codes previously generated by the trial statistician.  All project staff were unaware of group assignments until the completion of the trial and after data analysis. \| \| Blinding of participants and  personnel (performance bias) \| Low \| Quote: Supplements were packaged into identical pots, each containing 180 capsules, and labeled by staff who were not involved in the study. \| \| Blinding of outcome  assessment (detection bias) \| Low \| Quote: Supplements were packaged into identical pots, each containing 180 capsules, and labeled by staff who were not involved in the study. \| \| Incomplete outcome data  (attrition bias) \| Low \| Quote: Withdrawal from the trial (49 individuals in the fish-oil group compared with 53 individuals in the placebo group) and deaths (9 individuals in the fish-oil group compared with 8 individuals in the placebo group) over the 24 mo after randomization were similar between trial arms. A further 9 and 17 participants in the fish-oil and placebo arms, respectively, discontinued randomized treatment but provided data at the end of the trial. \| \| Selective reporting (reporting  bias) \| Low \| Quote: Study was registered at www.controlled-trials.com as ISRCTN 72331636. \| \| Other bias \| Low \| Comment: No other sourced of bias identified. \|   Geleijnse 2012   \| Bias \| Judge \| Support \| \| --- \| --- \| --- \| \| Random sequence generation  (selection bias) \| Low \| Quote: randomly assigned to different margarine spreads \| \| Allocation concealment  (selection bias) \| Low \| Quote: receive 8 blinded margarine tubs of 250 g at home every 12 weeks by cooled courier transport. \| \| Blinding of participants and  personnel (performance bias) \| Low \| Quote: The four different types of trial margarine (developed by Unilever, Vlaardingen, The Netherlands) were identical in taste, odor, texture, and color.  receive 8 blinded margarine tubs of 250 g at home every 12 weeks by cooled courier transport. \| \| Blinding of outcome  assessment (detection bias) \| Low \| Quote: Data analysis will be carried out by an independent statistician (E.B.), who is also a member of the Data Safety and Monitoring Board. \| \| Incomplete outcome data  (attrition bias) \| Unclear \| Comment: The rate of attrition was comparable \| \| Selective reporting (reporting  bias) \| Low \| Quote: Trial registration: ClinicalTrials.gov number, NCT00127452 (http://clinicaltrials.gov). \| \| Other bias \| Low \| Comment: No other sources of bias identified. \|   Hashimoto 2016   \| Bias \| Judge \| Support \| \| --- \| --- \| --- \| \| Random sequence generation  (selection bias) \| Low \| Quote: participants from each unit, floor or division were randomly allocated to one of two groups – control or intervention. \| \| Allocation concealment  (selection bias) \| Low \| Quote: All study participants were blinded to the food products, as all participant meals were cooked by staff in the kitchen of the care facility or nursing home. \| \| Blinding of participants and  personnel (performance bias) \| Low \| Quote: All study participants were blinded to the food products, as all participant meals were cooked by staff in the kitchen of the care facility or nursing home. \| \| Blinding of outcome  assessment (detection bias) \| Unclear \| No information.  Quote: These scales were self-administered, with possible assistance from neuropsychologists and/or hospital nurses if necessary. \| \| Incomplete outcome data  (attrition bias) \| Low \| Comment: The rate of attrition was comparable. \| \| Selective reporting (reporting  bias) \| Unclear \| No information. \| \| Other bias \| Low \| Comment: No other sources of bias identified. \|   Hashimoto 2021   \| Bias \| Judge \| Support for judgement \| \| --- \| --- \| --- \| \| Random sequence generation  (selection bias) \| Low \| Quote: Seventy-five participants (40 women and 35 men) were randomly allocated to either the control group or the PO group. \| \| Allocation concealment  (selection bias) \| Unclear \| Comment: No information \| \| Blinding of participants and  personnel (performance bias) \| Unclear \| Comment: No information \| \| Blinding of outcome  assessment (detection bias) \| High \| Comment: single-blind  Fourth, although the group assignment and the type of oil distributed were not disclosed to the subjects, they may have been able to distinguish the type of oil by taste and odor. \| \| Incomplete outcome data  (attrition bias) \| Unclear \| Quote: The 12-month study was completed by 24 control group subjects and 35 PO group subjects (Figure 1). \| \| Selective reporting (reporting  bias) \| Unclear \| Comment: No information \| \| Other bias \| Low \| Comment: No other sources of bias identified. \|   Howe 2018   \| Bias \| Judge \| Support \| \| --- \| --- \| --- \| \| Random sequence generation  (selection bias) \| Low \| Quote: The first participant was randomly allocated by a coin toss. \| \| Allocation concealment  (selection bias) \| Low \| Quote: Their designated supplement (see below) was then dispensed for daily consumption over the next 10 weeks……. To ensure well-balanced treatment groups, participants were assigned to EPAX or placebo according to Altman’s allocation by minimization method based on their age and BP obtained at the screening visit. \| \| Blinding of participants and  personnel (performance bias) \| Low \| Quote: Blinding was maintained until all data analysis had been completed. \| \| Blinding of outcome  assessment (detection bias) \| Low \| Quote: Blinding was maintained until all data analysis had been completed. \| \| Incomplete outcome data  (attrition bias) \| Low \| Comment: Despite the disparity in dropout rates between the placebo and intervention groups, with 3 participants discontinuing in the former compared to none in the latter, it appears improbable that this discrepancy significantly influenced the overall findings. \| \| Selective reporting (reporting  bias) \| Low \| Quote: registration on the Australian Clinical Trials Register (ACTRN12614000762651). \| \| Other bias \| Low \| Comment: No other sources of bias identified. \|   Ichinose 2021   \| Bias \| Judge \| Support for judgement \| \| --- \| --- \| --- \| \| Random sequence generation  (selection bias) \| Low \| Quote: 87 participants were randomly divided into two groups: Group allocation was performed by stratified random assignment according to age and sex, as described previously. \| \| Allocation concealment  (selection bias) \| Low \| Quote: Group allocation was performed by stratified random assignment according to age and sex, as described previously. \| \| Blinding of participants and  personnel (performance bias) \| Low \| Quote: Neither the participants nor the researchers knew which milk beverages were being consumed.  The placebo and DHA-enriched milk beverages had no differences in appearance, flavor, or aroma. \| \| Blinding of outcome  assessment (detection bias) \| Low \| Quote: Neither the participants nor the researchers knew which milk beverages were being consumed. \| \| Incomplete outcome data  (attrition bias) \| Low \| Comment: comparable drop-out rates for two arms \| \| Selective reporting (reporting  bias) \| Low \| Quote: This study is registered in the UMIN Clinical Trial Registry（registry number UMIN000031699）. \| \| Other bias \| Low \| Comment: No other sources of bias identified. \|   Kuszewski 2020   \| Bias \| Judge \| Support \| \| --- \| --- \| --- \| \| Random sequence generation  (selection bias) \| Low \| Quote: were randomly assigned to 1 of the 4 treatment groups by Altman’s minimization method (22) by an independent investigator based on their age, BMI, and sex: \| \| Allocation concealment  (selection bias) \| Low \| Quote: were randomly assigned to 1 of the 4 treatment groups by Altman’s minimization method (22) by an independent investigator based on their age, BMI, and sex: \| \| Blinding of participants and  personnel (performance bias) \| Low \| Quote: The fish oil (Blackmores Omega BrainTM) and curcumin capsules (Blackmores Brain ActiveTM) were supplied by Blackmores Institute (Sydney, Australia) and were identical in appearance to their respective placebos, identifiable only by code numbers.  Blinding was maintained until all data analysis had been completed. \| \| Blinding of outcome  assessment (detection bias) \| Low \| Quote: Blinding was maintained until all data analysis had been completed. \| \| Incomplete outcome data  (attrition bias) \| Low \| Comment: Comparable drop-out rate between groups. \| \| Selective reporting (reporting  bias) \| Low \| Quote: This trial was registered at the Australian and New Zealand Clinical Trial Register as ACTRN12616000732482p.  Postintervention data (absolute changes) were analyzed using a per-protocol analysis \| \| Other bias \| Low \| Comment: No other sources of bias identified. \|   Lee 2013   \| Bias \| Judge \| Support \| \| --- \| --- \| --- \| \| Random sequence generation  (selection bias) \| Low \| Quote: Randomization was achieved using computer-generated random numbers in stratified permuted blocks of size four. \| \| Allocation concealment  (selection bias) \| Unclear \| No information \| \| Blinding of participants and  personnel (performance bias) \| Low \| Quote: Both the EPAX 1050TG and placebo were visually identical and odourless. \| \| Blinding of outcome  assessment (detection bias) \| Low \| Quote: The treatment code was released once data analyses have been completed. \| \| Incomplete outcome data  (attrition bias) \| Low \| Comment: Comparable drop-out rates between groups. \| \| Selective reporting (reporting  bias) \| Unclear \| No information \| \| Other bias \| Low \| Comment: No other sources of bias identified. \|   Macpherson 2022   \| Bias \| Judge \| Support \| \| --- \| --- \| --- \| \| Random sequence generation  (selection bias) \| Low \| Quote: Participants were randomized using a simple randomization, computer-generated, random sampling set, with an allocation ratio of 1:1, stratified for sex and age (60–69.9 years, 70–79.9 years, and 80–85.9 years), conducted by personnel not otherwise involved in the trial. \| \| Allocation concealment  (selection bias) \| Low \| Quote: Participants were provided with the supplement in non-transparent sachets and given details of the assigned exercise program.  To maintain double-blinding, participants and  trainers delivering the exercise were not informed that all individuals assigned to the multimodal exercise program would receive the active supplement drink and those allocated to the stretching/flexibility condition would receive the placebo. \| \| Blinding of participants and  personnel (performance bias) \| Low \| Quote: Participants were provided with the supplement in non-transparent sachets and given details of the assigned exercise program. \| \| Blinding of outcome  assessment (detection bias) \| Low \| Quote: On completion of the baseline appointment, group allocation was provided to the researcher involved in assigning participants to the intervention. conducted by personnel not otherwise involved in the trial.  All outcomes were assessed by personnel blinded to the treatment/placebo groups, except for the operator of the 6-month dual-energy X-ray absorptiometry (DXA) scan.  Analysis ofDXA scans were conducted by a researcher (RLD) blinded to group assignment.  Analysis ofprimary outcomes was completed by personnel blinded to the intervention assignment. \| \| Incomplete outcome data  (attrition bias) \| Low \| Comment: The dropout rates in both groups were comparable. \| \| Selective reporting (reporting  bias) \| Low \| Quote: The study trial protocol paper containing the trial details has been published previously [24]. The study was prospectively registered (ACTRN12616001549415). \| \| Other bias \| Low \| Comment: No other sources of bias identified. \|   Mengelberg 2022   \| Bias \| Judge \| Support \| \| --- \| --- \| --- \| \| Random sequence generation  (selection bias) \| Low \| Quote: A paid research assistant generated a list of random participant numbers from Random.org, assigned participant numbers to capsule groups and labelled capsule bottles, and was the only person to know the grouping arrangements until the end of the testing. \| \| Allocation concealment  (selection bias) \| Low \| Quote: A paid research assistant generated a list of random participant numbers from Random.org, assigned participant numbers to capsule groups and labelled capsule bottles, and was the only person to know the grouping arrangements until the end of the testing. \| \| Blinding of participants and  personnel (performance bias) \| Low \| Quote: The two types of capsules were identical in size, colour and shape, and both contained vitamin E (1.15 mg of vitamin E 1300 IU/g per capsule) as an anti‐oxidant and orange oil (10 mg per capsule) to help mask the taste of the oils. \| \| Blinding of outcome  assessment (detection bias) \| Low \| Quote: A paid research assistant generated a list of random participant numbers from Random.org, assigned participant numbers to capsule groups and labelled capsule bottles, and was the only person to know the grouping arrangements until the end of the testing. \| \| Incomplete outcome data  (attrition bias) \| Low \| Comment: Dropout rates for both groups were comparable. \| \| Selective reporting (reporting  bias) \| Low \| Quote: The trial was registered with the Australian New Zealand Clinical Trials Registry (www.anzctr.org.au; request number: 366439). \| \| Other bias \| Low \| Comment: No other sources of bias identified. \|   Ogawa 2023   \| Bias \| Judge \| Support \| \| --- \| --- \| --- \| \| Random sequence generation  (selection bias) \| Low \| Quote: The test sample allocation table was generated randomly, and study subjects were assigned. \| \| Allocation concealment  (selection bias) \| Low \| Quote: The study was blinded to both participants and researchers. \| \| Blinding of participants and  personnel (performance bias) \| Low \| Quote: The study was blinded to both participants and researchers.  Flaxseed oil was provided by NIPPN Corporation (Tokyo, Japan), and corn oil was  purchased from J-OIL MILLS, Inc. (Tokyo, Japan), and both were individually packed without any labels. The packages were completely identical in color, shape, and size between the test foods. \| \| Blinding of outcome  assessment (detection bias) \| Low \| Quote: The study was blinded to both participants and researchers.  All tests proceeded individually with two trained testers, who are research assistants at the Tohoku University (one tester for screening procedure, another for assessments pre-, after 6 and 12 weeks of intervention). \| \| Incomplete outcome data  (attrition bias) \| Low \| Quote: There was no exclusion or dropout during the study, therefore, all participants who were selected during the screening were entered in the statistical analysis. \| \| Selective reporting (reporting  bias) \| Low \| Quote: This RCT was registered at the University Hospital medical information network (UNIM) Clinical Trial Registry (UMIN000039901). \| \| Other bias \| Low \| Comment: No other sources of bias identified. \|   Power 2022   \| Bias \| Judge \| Support \| \| --- \| --- \| --- \| \| Random sequence generation  (selection bias) \| Low \| Quote: Eligible individuals were assigned to the active or placebo group using block randomisation with no stratification. Random allocation sequencing in block sizes of10 and in a 1:1 randomisation ratio was performed using a trial management system (Trial Controller) designed by our research centre and overseen by a Statistician (JS). \| \| Allocation concealment  (selection bias) \| Low \| Quote: Comprehensive security and access controls in relation to the storage of the electronic data and the prevention of unauthorised access were implemented for this software.  Capsule dispensing was performed by members (CK and LOB) of  UPMC Whitfield Pharmacy, Waterford, Ireland. Using the Trial Controller system these individuals had access to patient study codes, assigned intervention group and capsule batch numbers. Importantly, pharmacy members had no contact with participants and no access to participant names or contact details. By comparison, researchers directly involved in CARES had access to participant details and study codes, but no access to information regarding intervention allocation or capsule batch numbers. The researcher (RP) received a box of tablets from the pharmacy members (CK or LOB) with a subject identification label (i.e. both the researcher and study participant were blinded to the intervention). The intervention code was only revealed at study completion. \| \| Blinding of participants and  personnel (performance bias) \| Low \| Quote: Active and placebo capsules were identical in colour and size. Each active capsule contained equal quantities of fish oil, carotenoids and vitamin E. \| \| Blinding of outcome  assessment (detection bias) \| Low \| Quote: Comprehensive security and access controls in relation to the storage of the electronic data and the prevention of unauthorised access were implemented for this software. \| \| Incomplete outcome data  (attrition bias) \| Low \| Comment: The dropout rates for both groups were comparable. \| \| Selective reporting (reporting  bias) \| Low \| Quote: CARES (trial registration number: ISRCTN10431469) \| \| Other bias \| Low \| Comment: No other sources of bias identified. \|   **Sala-Vila, 2020**   \| Bias \| Judge \| Support \| \| --- \| --- \| --- \| \| Random sequence generation  (selection bias) \| Low \| Quote: We randomly assigned participants to either the walnut group (consuming ∼15% of daily energy intake as walnuts) or the control group (abstention from walnuts) using a computerized, web-based, random number table with stratification by gender  and age range in a 1:1 ratio in each center. \| \| Allocation concealment  (selection bias) \| High \| Quote: All study clinicians and researchers were blind to participants’ intervention group, except for the dietitians in charge of dietary assessment and walnut supply. \| \| Blinding of participants and  personnel (performance bias) \| High \| Quote: participants were not blinded to the intervention, because it consisted of a whole food \| \| Blinding of outcome  assessment (detection bias) \| Low \| Quote: All data obtained during the study were recorded in a dedicated online database developed by Costaisa SA. An independent Data and Safety Monitoring Board blinded to subject allocation periodically reviewed and evaluated the accumulated study data during the trial’s progress. \| \| Incomplete outcome data  (attrition bias) \| Unclear \| Comment: No information \| \| Selective reporting (reporting  bias) \| Low \| Quote: This trial was registered at clinicaltrials.gov as NCT01634841. \| \| Other bias \| Low \| Comment: No other sources of bias identified \|   Sinn 2012   \| Bias \| Judge \| Support \| \| --- \| --- \| --- \| \| Random sequence generation  (selection bias) \| Low \| Quote: Invited to take part and block randomised  to treatment \| \| Allocation concealment  (selection bias) \| Unclear \| Comment: No information \| \| Blinding of participants and  personnel (performance bias) \| Low \| Quote: All researchers involved with participants, data entry or analysis and participants were blinded to treatment conditions \| \| Blinding of outcome  assessment (detection bias) \| Low \| Quote: All researchers involved with participants, data entry or analysis and participants were blinded to treatment conditions \| \| Incomplete outcome data  (attrition bias) \| Low \| Quote: The number of dropouts between the groups was not significantly different (P=0.39) \| \| Selective reporting (reporting  bias) \| Unclear \| Comment: No information \| \| Other bias \| Low \| Comment: No other sources of bias identified \|   **Tokuda 2020**   \| Bias \| Judge \| Support \| \| --- \| --- \| --- \| \| Random sequence generation  (selection bias) \| Low \| Quote: Enrolled participants were randomly assigned in a 1:1:1 ratio based on dynamic allocation to achieve balance among the groups regarding age, sex, WMS-R LM II, ARA and DHA composition in plasma phospholipids by using a spread sheet program with RAND function of Microsoft Excel 2013. \| \| Allocation concealment  (selection bias) \| Low \| Quote: The randomisation procedure was performed by a person who was not involved in this study. Following, the randomisation codes for these participants and the codes for masked supplements were each held in sealed opaque envelopes by 2 different individuals who were uninvolved in this study. Information about these assignments was masked to researchers until all data were collected and analysed. \| \| Blinding of participants and  personnel (performance bias) \| Low \| Quote: Capsules of LCPUFA and placebo were same size and colour. this study design was single blinded RCT, participants were blinded to the supplementation (the placebo or the LCPUFA) in the exercise groups \| \| Blinding of outcome  assessment (detection bias) \| Low \| Quote: Researchers involved in the assessment of outcome measures were blinded to the randomisation assignment. \| \| Incomplete outcome data  (attrition bias) \| Unclear \| No information \| \| Selective reporting (reporting  bias) \| Low \| Quote: This study was registered in the University Hospital Medical Information Network (UMIN) Clinical Trial Registry (UMIN000030065) \| \| Other bias \| Low \| Comment: No other sources of bias identified. \|   **Valls-Pedret 2015**   \| Bias \| Judge \| Support \| \| --- \| --- \| --- \| \| Random sequence generation  (selection bias) \| Low \| Quote: Randomization was performed centrally by  means of a computer-generated random-number sequence in blocks of 50 participants balanced by sex and age \| \| Allocation concealment  (selection bias) \| Unclear \| No information \| \| Blinding of participants and  personnel (performance bias) \| Unclear \| No information \| \| Blinding of outcome  assessment (detection bias) \| Low \| Quote: Outcomes are ascertained on a yearly basis by a Clinical Events Committee whose members are blinded to the intervention group. \| \| Incomplete outcome data  (attrition bias) \| High \| Quote: Dropouts were unevenly distributed among groups (18.1% Mediterranean diet plus olive oil, 28.8% Mediterranean diet plus nuts, and 34.5%  control diet; *P* = .004).  Nevertheless, dropout rates per group followed a pattern similar to that of the main trial and were not unexpected given the older age of participants and the trial’s duration. \| \| Selective reporting (reporting  bias) \| Low \| Quote: The trial is registered (http://www.controlled-trials.com/ISRCTN35739639). \| \| Other bias \| Low \| Comment: No other sources of bias identified. \|   **van de Rest 2008**   \| Bias \| Judge \| Support \| \| --- \| --- \| --- \| \| Random sequence generation  (selection bias) \| Low \| Quote: An independent person randomized subjects by means of computer-generated random numbers in stratified permuted blocks of six. \| \| Allocation concealment  (selection bias) \| Low \| Quote: Capsules with fish oil or placebo oil were indistinguishable in appearance. Staff members and participants were blinded toward treatment allocation until completion of blind data analysis. \| \| Blinding of participants and  personnel (performance bias) \| Low \| Quote: Capsules with fish oil or placebo oil were indistinguishable in appearance. Staff members and participants were blinded toward treatment allocation until completion of blind data analysis.  At the end of the study, blinding of subjects toward treatment allocation (fish oil, placebo, or no idea) was evaluated. The proportion of participants who thought they had received fish oil or placebo did not differ among the groups. \| \| Blinding of outcome  assessment (detection bias) \| Low \| Quote: Staff members and participants were blinded toward treatment allocation until completion of blind data analysis \| \| Incomplete outcome data  (attrition bias) \| Low \| Comment: Apart from the individuals who stopped treatment prematurely, the average adherence to treatments based on counts of returned capsules was high (99%, with only three subjects < 80%) and did not differ among the treatment groups. \| \| Selective reporting (reporting  bias) \| Unclear \| Comment: No information \| \| Other bias \| Low \| Comment: No other sources of bias identified \|   **Yurko-Mauro 2010**   \| Bias \| Judge \| Support \| \| --- \| --- \| --- \| \| Random sequence generation  (selection bias) \| Low \| Quote: Eligible subjects were stratified by age (55–69; R70) and randomized 1:1 in blocks of four to active or placebo by site, using a centralized interactive voice randomization system (Fisher Clinical, FACTS services, Allentown, PA). \| \| Allocation concealment  (selection bias) \| Unclear \| Comment: No information \| \| Blinding of participants and  personnel (performance bias) \| Low \| Quote: All capsules were orange-flavored and orange color to protect the study blind \| \| Blinding of outcome  assessment (detection bias) \| Low \| Quote: A preplanned interim analysis (IA) for futility was conducted by an unblinded statistician not associated with data collection after 140 subjects completed the study \| \| Incomplete outcome data  (attrition bias) \| Low \| Comment: Comparable drop-out rates between groups. \| \| Selective reporting (reporting  bias) \| Low \| Quote: Clinicaltrials.gov, Identifier: NCT0027813. \| \| Other bias \| Low \| Comment: No other sources of bias identified \| |
| --- | --- | --- | --- | --- | --- | --- | --- | --- | --- | --- | --- | --- | --- | --- | --- | --- | --- | --- | --- | --- | --- | --- | --- | --- | --- | --- | --- | --- | --- | --- | --- | --- | --- | --- | --- | --- | --- | --- | --- | --- | --- | --- | --- | --- | --- | --- | --- | --- | --- | --- | --- | --- | --- | --- | --- | --- | --- | --- | --- | --- | --- | --- | --- | --- | --- | --- | --- | --- | --- | --- | --- | --- | --- | --- | --- | --- | --- | --- | --- | --- | --- | --- | --- | --- | --- | --- | --- | --- | --- | --- | --- | --- | --- | --- | --- | --- | --- | --- | --- | --- | --- | --- | --- | --- | --- | --- | --- | --- | --- | --- | --- | --- | --- | --- | --- | --- | --- | --- | --- | --- | --- | --- | --- | --- | --- | --- | --- | --- | --- | --- | --- | --- | --- | --- | --- | --- | --- | --- | --- | --- | --- | --- | --- | --- | --- | --- | --- | --- | --- | --- | --- | --- | --- | --- | --- | --- | --- | --- | --- | --- | --- | --- | --- | --- | --- | --- | --- | --- | --- | --- | --- | --- | --- | --- | --- | --- | --- | --- | --- | --- | --- | --- | --- | --- | --- | --- | --- | --- | --- | --- | --- | --- | --- | --- | --- | --- | --- | --- | --- | --- | --- | --- | --- | --- | --- | --- | --- | --- | --- | --- | --- | --- | --- | --- | --- | --- | --- | --- | --- | --- | --- | --- | --- | --- | --- | --- | --- | --- | --- | --- | --- | --- | --- | --- | --- | --- | --- | --- | --- | --- | --- | --- | --- | --- | --- | --- | --- | --- | --- | --- | --- | --- | --- | --- | --- | --- | --- | --- | --- | --- | --- | --- | --- | --- | --- | --- | --- | --- | --- | --- | --- | --- | --- | --- | --- | --- | --- | --- | --- | --- | --- | --- | --- | --- | --- | --- | --- | --- | --- | --- | --- | --- | --- | --- | --- | --- | --- | --- | --- | --- | --- | --- | --- | --- | --- | --- | --- | --- | --- | --- | --- | --- | --- | --- | --- | --- | --- | --- | --- | --- | --- | --- | --- | --- | --- | --- | --- | --- | --- | --- | --- | --- | --- | --- | --- | --- | --- | --- | --- | --- | --- | --- | --- | --- | --- | --- | --- | --- | --- | --- | --- | --- | --- | --- | --- | --- | --- | --- | --- | --- | --- | --- | --- | --- | --- | --- | --- | --- | --- | --- | --- | --- | --- | --- | --- | --- | --- | --- | --- | --- | --- | --- | --- | --- | --- | --- | --- | --- | --- | --- | --- | --- | --- | --- | --- | --- | --- | --- | --- | --- | --- | --- | --- | --- | --- | --- | --- | --- | --- | --- | --- | --- | --- | --- | --- | --- | --- | --- | --- | --- | --- | --- | --- | --- | --- | --- | --- | --- | --- | --- | --- | --- | --- | --- | --- | --- | --- | --- | --- | --- | --- | --- | --- | --- | --- | --- | --- | --- | --- | --- | --- | --- | --- | --- | --- | --- | --- | --- | --- | --- | --- | --- | --- | --- | --- | --- | --- | --- | --- | --- | --- | --- | --- | --- | --- | --- | --- | --- | --- | --- | --- | --- | --- | --- | --- | --- | --- | --- | --- | --- | --- | --- | --- | --- | --- | --- | --- | --- | --- | --- | --- | --- | --- | --- | --- | --- | --- | --- | --- | --- | --- | --- | --- | --- | --- | --- | --- | --- | --- | --- | --- | --- | --- | --- | --- | --- | --- | --- | --- | --- | --- | --- | --- | --- | --- | --- | --- | --- | --- | --- | --- | --- | --- | --- | --- | --- | --- | --- | --- | --- | --- | --- | --- | --- | --- | --- | --- | --- | --- | --- | --- | --- | --- | --- | --- | --- | --- | --- | --- | --- | --- | --- | --- | --- | --- | --- |

Table S4. Sensitivity analyses for each of the six cognitive domains.

| Cognitive domain  examined | Omitted study | Summary SMD (95% CI) | I^2^ (%) |
| --- | --- | --- | --- |
| Global cognition |  | 0.04 (-0.11, 0.19) | 41 |
|  | Andrieu 2017 | 0.05 (-0.12, 0.21) | 45 |
|  | Arellanes 2020 | 0.05 (-0.10, 0.21) | 44 |
|  | Bischoff-Ferrari 2020 | 0.04 (-0.12, 0.21) | 44 |
|  | **Bo 2017** | **-0.01 (-0.08, 0.06)** | **0** |
|  | Chew 2015 | 0.05 (-0.12, 0.21) | 45 |
|  | Dangour 2010 | 0.05 (-0.11, 0.21) | 45 |
|  | Geleijnse 2012 | 0.04 (-0.12, 0.21) | 45 |
|  | Hahimoto 2016 | 0.04 (-0.12, 0.20) | 45 |
|  | Howe 2018 | 0.04 (-0.12, 0.20) | 45 |
|  | Ichinose 2021 | 0.01 (-0.12, 0.15) | 34 |
|  | Macpherson 2022 | 0.05 (-0.11, 0.21) | 45 |
|  | Mengelberg 2022 | 0.03 (-0.12, 0.19) | 44 |
|  | Ogawa 2023 | 0.04 (-0.12, 0.20) | 45 |
|  | Power 2022 | 0.05 (-0.10, 0.21) | 43 |
|  | Valls-Pedret 2015 | 0.06 (-0.10, 0.22) | 40 |
|  | Yurko-Mauro 2010 | 0.05 (-0.11, 0.21) | 44 |
|  |  | | |
| Episodic memory |  | 0.06 (-0.09, 0.22) | 37 |
|  | Andrieu 2017 | 0.09 (-0.08, 0.26) | 24 |
|  | Arellanes 2020 | 0.06 (-0.11, 0.23) | 41 |
|  | Bo 2017 | 0.08 (-0.09, 0.25) | 41 |
|  | Dadova 2022 | 0.06 (-0.11, 0.23) | 40 |
|  | Dangour 2010 | 0.07 (-0.11, 0.25) | 41 |
|  | Kuszewski 2020 | 0.08 (-0.09, 0.25) | 40 |
|  | **Lee 2013** | **0.02 (-0.07, 0.10)** | **0** |
|  | Mengelberg 2022 | 0.06 (-0.11, 0.23) | 40 |
|  | Ogawa 2023 | 0.09 (-0.07, 0.24) | 34 |
|  | Sala-Vila 2020 | 0.08 (-0.10, 0.25) | 40 |
|  | Tokuda 2020 | 0.06 (-0.11, 0.23) | 40 |
|  | Valls-Pedret 2015 | 0.06 (-0.12, 0.24) | 39 |
|  | van de Rest 2008 | 0.07 (-0.11, 0.24) | 41 |
|  | Yurko-Mauro 2010 | 0.06 (-0.12, 0.24) | 37 |
|  |  | | |
| Executive function |  | 0.28 (-0.07, 0.62) | 74 |
|  | Andrieu 2017 | 0.30 (-0.07, 0.67) | 75 |
|  | Arellanes 2020 | 0.30 (-0.07, 0.67) | 75 |
|  | Bo 2017 | 0.27 (-0.10, 0.64) | 74 |
|  | Dangour 2010 | 0.30 (-0.07, 0.67) | 75 |
|  | Hashimoto 2021 | 0.27 (-0.10, 0.64) | 74 |
|  | Howe 2018 | 0.24 (-0.12, 0.60) | 72 |
|  | Kuszewski 2020 | 0.31 (-0.06, 0.67) | 75 |
|  | Lee 2013 | 0.24 (-0.12, 0.60) | 72 |
|  | Macpherson 2022 | 0.31 (-0.06, 0.67) | 75 |
|  | Mengelberg 2022 | 0.30 (-0.07, 0.67) | 75 |
|  | Ogawa 2023 | 0.27 (-0.10, 0.64) | 73 |
|  | Power 2022 | 0.25 (-0.11, 0.62) | 73 |
|  | Sala-Vila 2020 | 0.30 (-0.07, 0.67) | 75 |
|  | **Sinn 2012** | **0.13 (-0.06, 0.31)** | **56** |
|  | Tokuda 2020 | 0.29 (-0.08, 0.66) | 75 |
|  | Valls-Pedret 2015 | 0.31 (-0.05, 0.68) | 74 |
|  | van de Rest 2008 | 0.30 (-0.06, 0.67) | 75 |
|  | Yurko-Mauro 2010 | 0.30 (-0.07, 0.67) | 75 |
|  |  | | |
| Processing speed |  | 0.24 (-0.14, 0.62) | 72 |
|  | Andrieu 2017 | 0.28 (-0.16, 0.72) | 75 |
|  | Bo 2017 | 0.17 (-0.22, 0.56) | 63 |
|  | Dangour 2010 | 0.28 (-0.16, 0.72) | 75 |
|  | **Howe 2018** | **0.10 (-0.14, 0.35)** | **47** |
|  | Lee 2013 | 0.27 (-0.16, 0.70) | 75 |
|  | Macpherson 2022 | 0.30 (-0.13, 0.72) | 74 |
|  | Ogawa 2023 | 0.26 (-0.18, 0.70) | 75 |
|  | Power 2022 | 0.23 (-0.21, 0.67) | 74 |
|  | Sala-Vila 2020 | 0.28 (-0.15, 0.72) | 74 |
|  |  | | |
| Attention |  | 0.05 (-0.06, 0.16) | 13 |
|  | Andrieu 2017 | 0.02 (-0.10, 0.14) | 0 |
|  | Arellanes 2020 | 0.05 (-0.07, 0.17) | 22 |
|  | Dangour 2010 | 0.07 (-0.05, 0.19) | 0 |
|  | Macpherson 2022 | 0.05 (-0.08, 0.17) | 22 |
|  | Mengelberg 2022 | 0.06 (-0.05, 0.17) | 14 |
|  | Ogawa 2023 | 0.06 (-0.03, 0.16) | 4 |
|  | Sala-Vila 2020 | 0.05 (-0.08, 0.18) | 22 |
|  | Tokuda 2020 | 0.04 (-0.07, 0.15) | 18 |
|  | Valls-Pedret 2015 | 0.05 (-0.08, 0.18) | 22 |
|  | van de Rest 2008 | 0.02 (-0.08, 0.12) | 0 |
|  |  | | |
| Visuospatial function |  | 0.16 (-0.35, 0.66) | 71 |
|  | **Bo 2017** | **-0.03 (-0.36, 0.30)** | **0** |
|  | Lee 2013 | 0.16 (-0.57, 0.89) | 77 |
|  | Mengelberg 2022 | 0.14 (-0.60, 0.88) | 76 |
|  | Ogawa 2023 | 0.25 (-0.39, 0.88) | 77 |
|  | Sala-Vila 2020 | 0.26 (-0.44, 0.96) | 63 |
|  |  | | |

Figure S1. Dose-response meta-analyses for the association between n-3 polyunsaturated fatty acids (PUFA) and the episodic memory. (a) duration of intervention; (b) daily intake of n-3 PUFA; (c) total amount of n-3 PUFA taken during the study period; (d) daily intake of docosahexaenoic acid (DHA); (e) daily intake of eicosapentaenoic acid (EPA); (f) ratio of DHA to EPA taken.

Figure S2. Dose-response meta-analyses for the association between n-3 polyunsaturated fatty acids (PUFA) and the processing speed. (a) duration of intervention; (b) daily intake of n-3 PUFA; (c) total amount of n-3 PUFA taken during the study period; (d) daily intake of docosahexaenoic acid (DHA); (e) daily intake of eicosapentaenoic acid (EPA); (f) ratio of DHA to EPA taken.

Figure S3. Dose-response meta-analyses for the association between n-3 polyunsaturated fatty acids (PUFA) and the attention. (a) duration of intervention; (b) daily intake of n-3 PUFA; (c) total amount of n-3 PUFA taken during the study period; (d) daily intake of docosahexaenoic acid (DHA); (e) daily intake of eicosapentaenoic acid (EPA); (f) ratio of DHA to EPA taken.

Figure S4. Dose-response meta-analyses for the association between n-3 polyunsaturated fatty acids (PUFA) and the visuospatial function. (a) duration of intervention; (b) daily intake of n-3 PUFA; (c) total amount of n-3 PUFA taken during the study period; (d) daily intake of docosahexaenoic acid (DHA); (e) daily intake of eicosapentaenoic acid (EPA); (f) ratio of DHA to EPA taken.

Figure S5. Dose-response meta-analyses for the association between n-3 polyunsaturated fatty acids (PUFA) and the global cognition based on the studies from countries where the blood level of DHA + EPA is very low. (a) duration of intervention; (b) daily intake of n-3 PUFA; (c) total amount of n-3 PUFA taken during the study period; (d) daily intake of docosahexaenoic acid (DHA); (e) daily intake of eicosapentaenoic acid (EPA); (f) ratio of DHA to EPA taken.

Figure S6. Dose-response meta-analyses for the association between n-3 polyunsaturated fatty acids (PUFA) and the executive function based on the studies from countries where the blood level of DHA + EPA is very low. (a) duration of intervention; (b) daily intake of n-3 PUFA; (c) total amount of n-3 PUFA taken during the study period; (d) daily intake of docosahexaenoic acid (DHA); (e) daily intake of eicosapentaenoic acid (EPA); (f) ratio of DHA to EPA taken.

Figure S7. Dose-response meta-analyses for the association between n-3 polyunsaturated fatty acids (PUFA) and the global cognition based on the studies from countries where the blood level of DHA + EPA is not very low. (a) duration of intervention; (b) daily intake of n-3 PUFA; (c) total amount of n-3 PUFA taken during the study period; (d) daily intake of docosahexaenoic acid (DHA); (e) daily intake of eicosapentaenoic acid (EPA); (f) ratio of DHA to EPA taken.

Figure S8. Dose-response meta-analyses for the association between n-3 polyunsaturated fatty acids (PUFA) and the executive function of the people with mild cognitive impairment. (a) daily intake of n-3 PUFA; (b) total amount of n-3 PUFA taken during the study period; (c) daily intake of docosahexaenoic acid (DHA); (d) daily intake of eicosapentaenoic acid (EPA); (e) ratio of DHA to EPA taken.

*Due to the lack of data, it was not possible to conduct the analyses on the duration of intervention.

Figure S9. Dose-response meta-analyses for the association between n-3 polyunsaturated fatty acids (PUFA) and the global cognition of the cognitively normal individuals. (a) duration of intervention; (b) daily intake of n-3 PUFA; (c) total amount of n-3 PUFA taken during the study period; (d) daily intake of docosahexaenoic acid (DHA); (e) daily intake of eicosapentaenoic acid (EPA); (f) ratio of DHA to EPA taken.

Figure S10. Dose-response meta-analyses for the association between n-3 polyunsaturated fatty acids (PUFA) and the executive function of the cognitively normal individuals. (a) duration of intervention; (b) daily intake of n-3 PUFA; (c) total amount of n-3 PUFA taken during the study period; (d) daily intake of docosahexaenoic acid (DHA); (e) daily intake of eicosapentaenoic acid (EPA); (f) ratio of DHA to EPA taken.

Figure S11. Funnel plots of meta-analyses between n-3 polyunsaturated fatty acids and the (a) global cognition, (b) episodic memory, (c) executive function, (d) processing speed, (e) attention, and (f) visuospatial function.
