# Supplementary material for: The influence of n-3 polyunsaturated fatty acids on cognitive function in individuals without dementia: a systematic review and dose–response meta-analysis
Source: BMC Med. 2024 Mar 12;22:109. doi: 10.1186/s12916-024-03296-0 (PMC10929146; doi:10.1186/s12916-024-03296-0)
Supplement: Supplementary file 1 — Additional file 1: Table S1. Search strategies by data sources. Table S2. Cognitive tests employed in each trial by their corresponding cognitive domain. Table S3. Detailed description of the risk of bias (RoB) for the individual studies. Table S4. Sensitivity analyses for each of the six cognitive domains. Fig. S1. Dose-response meta-analyses for the association between n-3 polyunsaturated fatty acids (PUFA) and the episodic memory. (a) duration of intervention; (b) daily intake of n-3 PUFA; (c) total amount of n-3 PUFA taken during the study period; (d) daily intake of docosahexaenoic acid (DHA); (e) daily intake of eicosapentaenoic acid (EPA); (f) ratio of DHA to EPA taken. Fig. S2. Dose-response meta-analyses for the association between n-3 polyunsaturated fatty acids (PUFA) and the processing speed. (a) duration of intervention; (b) daily intake of n-3 PUFA; (c) total amount of n-3 PUFA taken during the study period; (d) daily intake of docosahexaenoic acid (DHA); (e) daily intake of eicosapentaenoic acid (EPA); (f) ratio of DHA to EPA taken. Fig. S3. Dose-response meta-analyses for the association between n-3 polyunsaturated fatty acids (PUFA) and the attention. (a) duration of intervention; (b) daily intake of n-3 PUFA; (c) total amount of n-3 PUFA taken during the study period; (d) daily intake of docosahexaenoic acid (DHA); (e) daily intake of eicosapentaenoic acid (EPA); (f) ratio of DHA to EPA taken. Fig. S4. Dose-response meta-analyses for the association between n-3 polyunsaturated fatty acids (PUFA) and the visuospatial function. (a) duration of intervention; (b) daily intake of n-3 PUFA; (c) total amount of n-3 PUFA taken during the study period; (d) daily intake of docosahexaenoic acid (DHA); (e) daily intake of eicosapentaenoic acid (EPA); (f) ratio of DHA to EPA taken. Fig. S5. Dose-response meta-analyses for the association between n-3 polyunsaturated fatty acids (PUFA) and the global cognition based on the studies from countries where the blood l [file 12916_2024_3296_MOESM1_ESM.zip › Results_240115.pptx]

## Slide 1
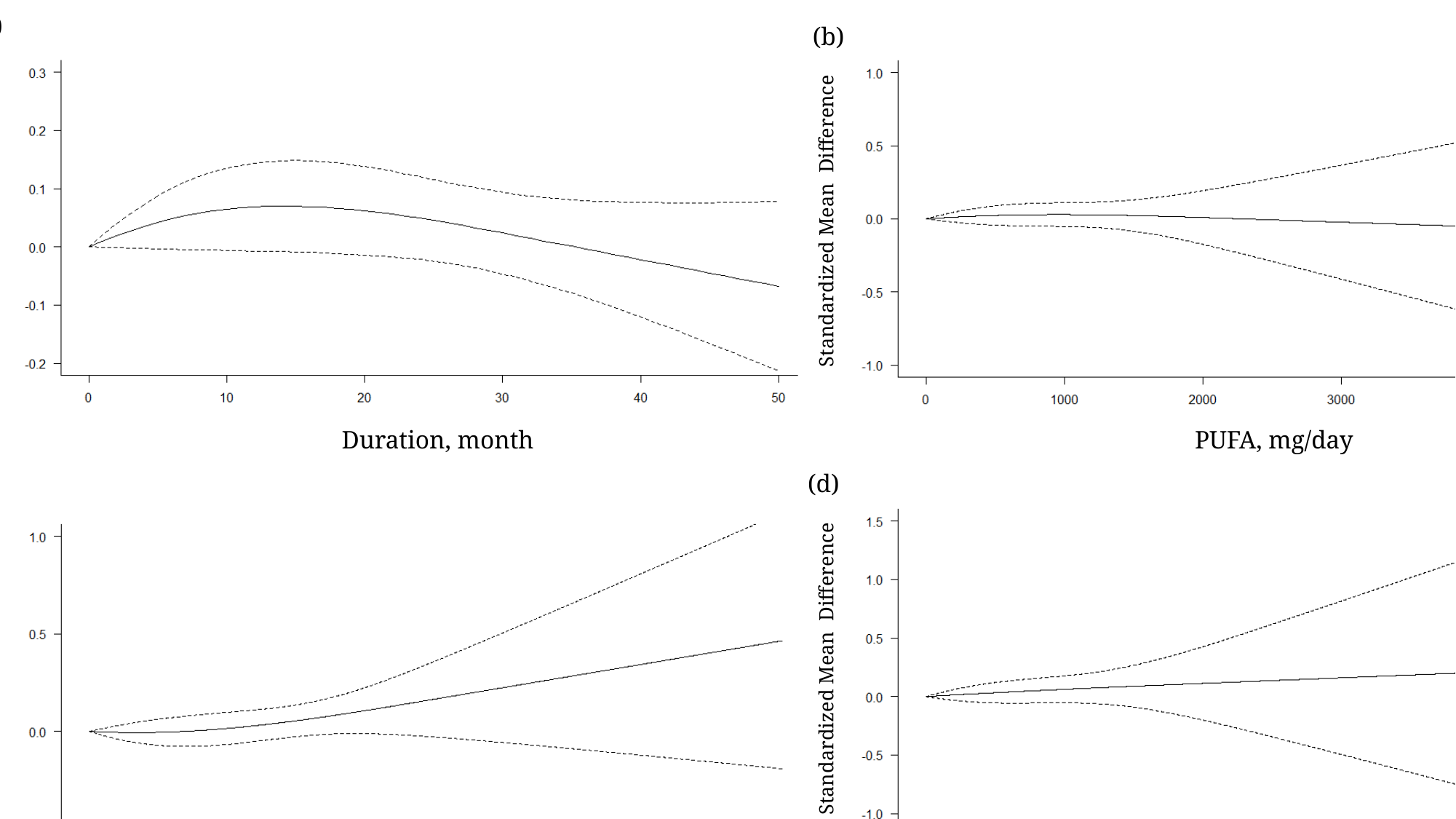

(a)
(b)
Standardized Mean Difference
Standardized Mean Difference
PUFA, mg/day
Duration, month
(d)
(c)
Standardized Mean Difference
Standardized Mean Difference
Total amount of PUFA, g
DHA, mg/day
(f)
(e)
Standardized Mean Difference
Standardized Mean Difference
EPA, mg/day
DHA/EPA ratio

## Slide 2
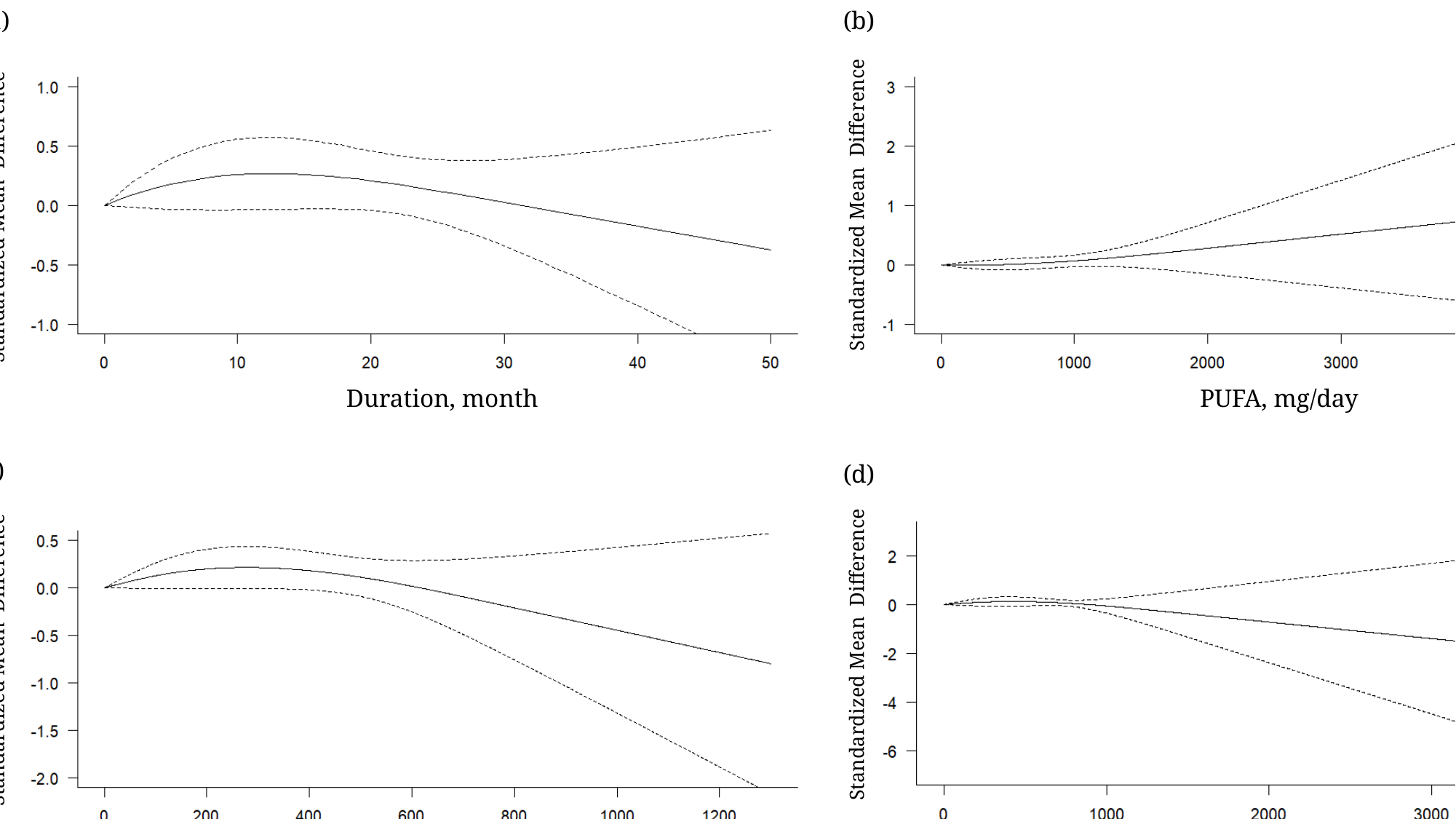

(a)
(b)
Standardized Mean Difference
Standardized Mean Difference
Duration, month
PUFA, mg/day
(c)
(d)
Standardized Mean Difference
Standardized Mean Difference
Total amount of PUFA, g
DHA, mg/day
(f)
(e)
Standardized Mean Difference
Standardized Mean Difference
EPA, mg/day
DHA/EPA ratio

## Slide 3
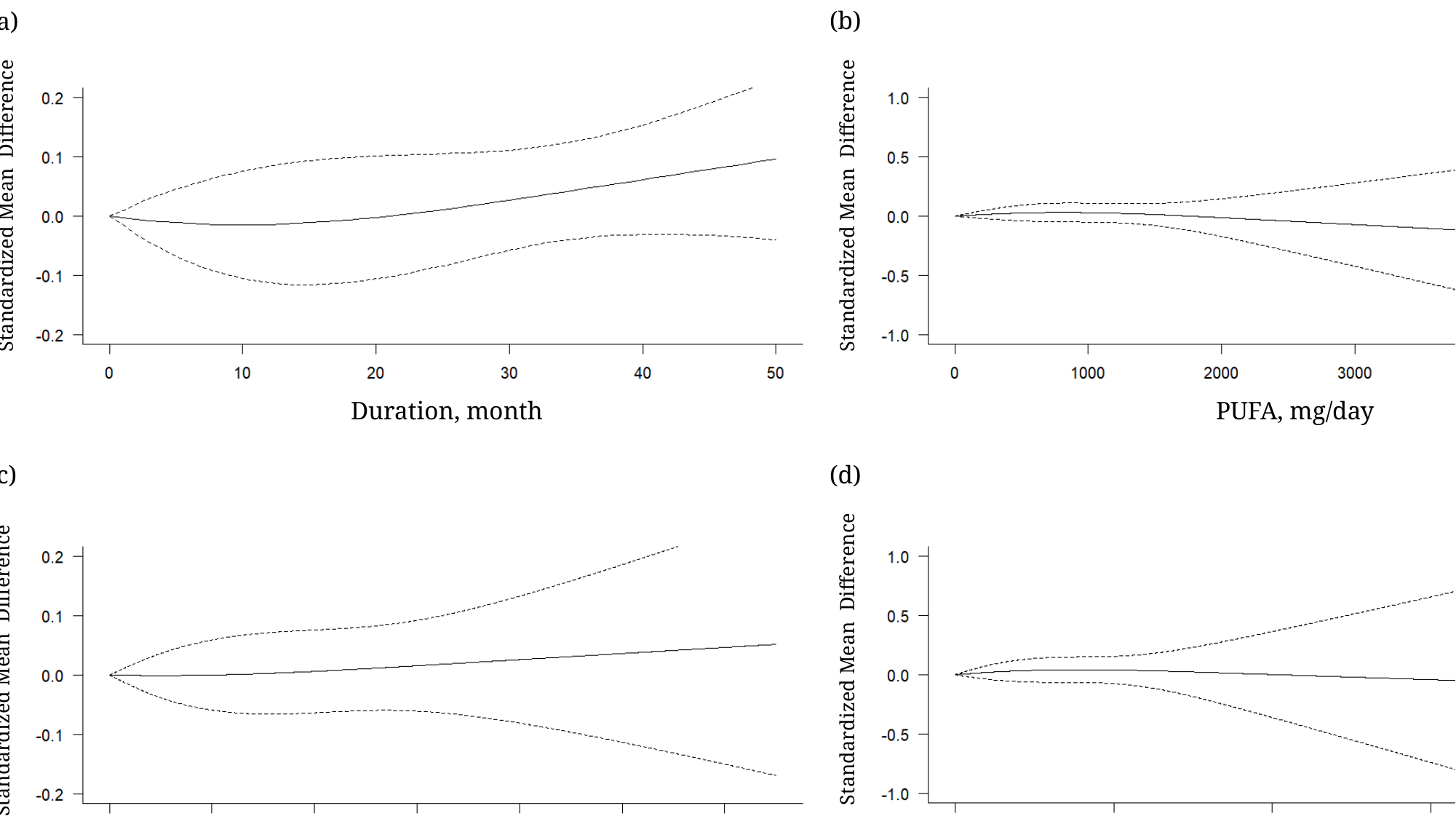

(b)
(a)
Standardized Mean Difference
Standardized Mean Difference
Duration, month
PUFA, mg/day
(c)
(d)
Standardized Mean Difference
Standardized Mean Difference
Total amount of PUFA, g
DHA, mg/day
(e)
(f)
Standardized Mean Difference
Standardized Mean Difference
DHA/EPA ratio
EPA, mg/day

## Slide 4
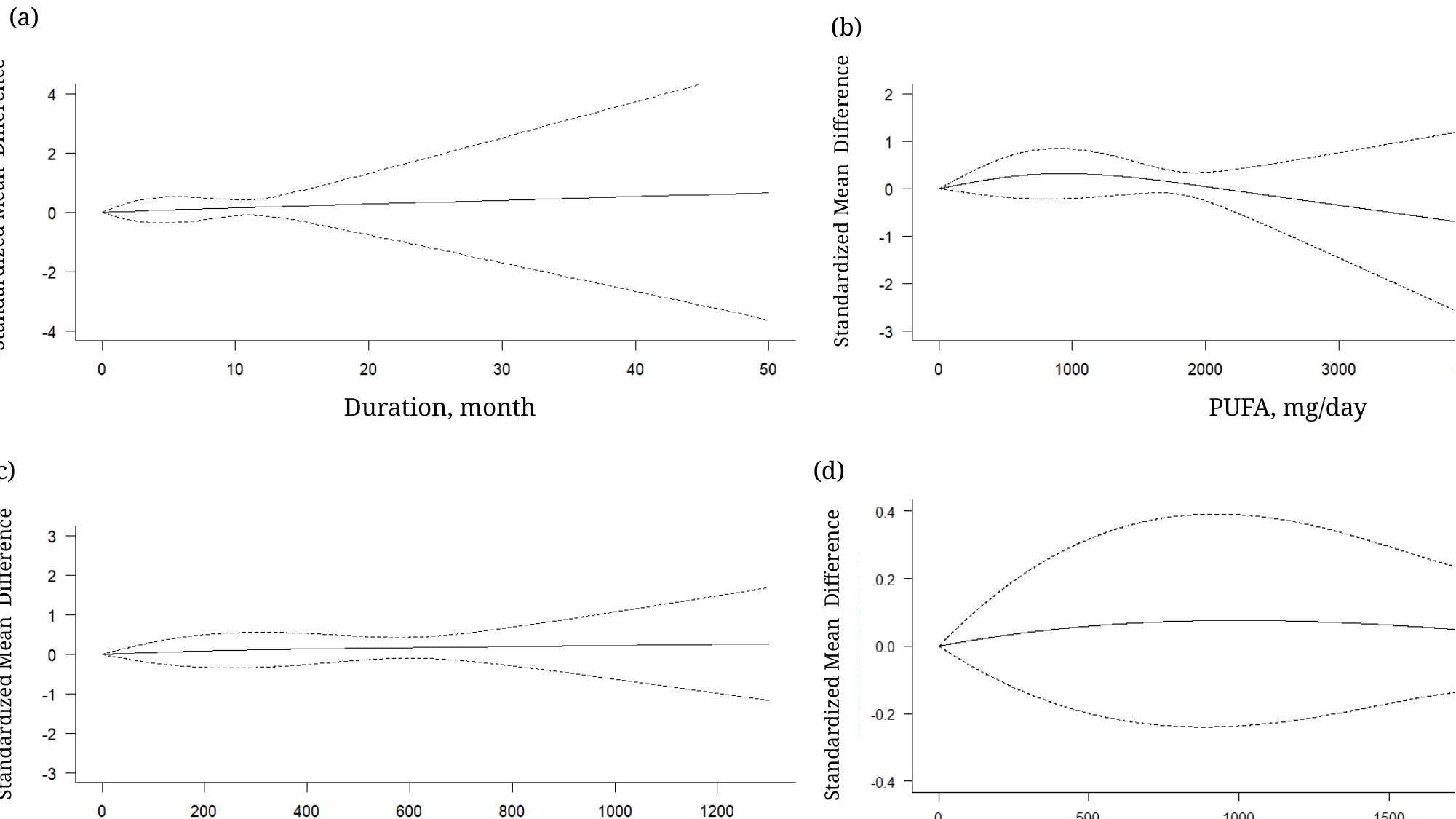

(b)
(a)
Standardized Mean Difference
Standardized Mean Difference
Duration, month
PUFA, mg/day
(c)
(d)
Standardized Mean Difference
Standardized Mean Difference
Total amount of PUFA, g
DHA, mg/day
(e)
(f)
Standardized Mean Difference
Standardized Mean Difference
EPA, mg/day
DHA/EPA ratio

## Slide 5
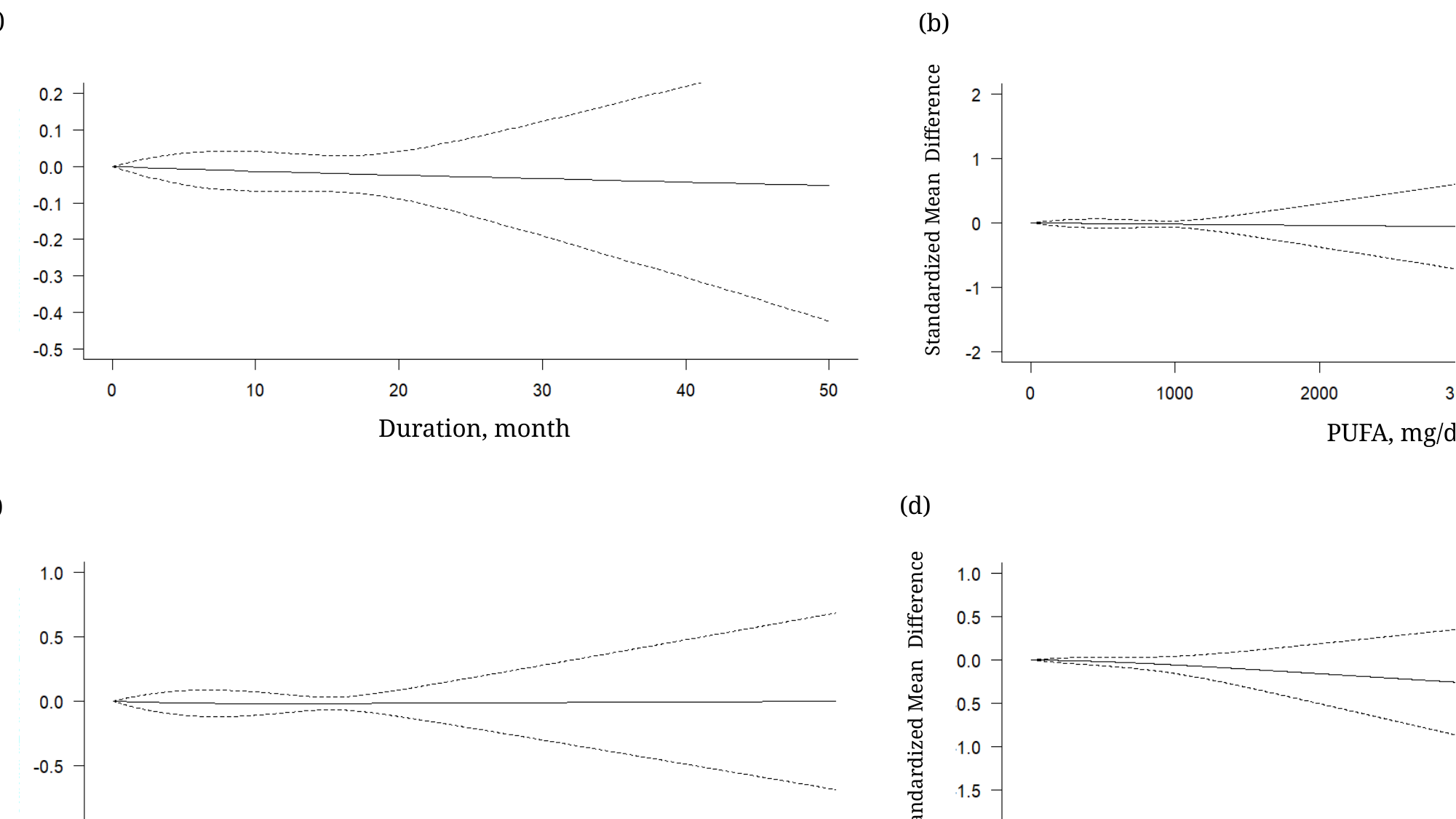

(a)
(b)
Standardized Mean Difference
Standardized Mean Difference
Duration, month
PUFA, mg/day
(d)
(c)
Standardized Mean Difference
Standardized Mean Difference
Standardized Mean Difference
Total amount of PUFA, g
DHA, mg/day
(f)
(e)
Standardized Mean Difference
Standardized Mean Difference
EPA, mg/day
DHA/EPA ratio

## Slide 6
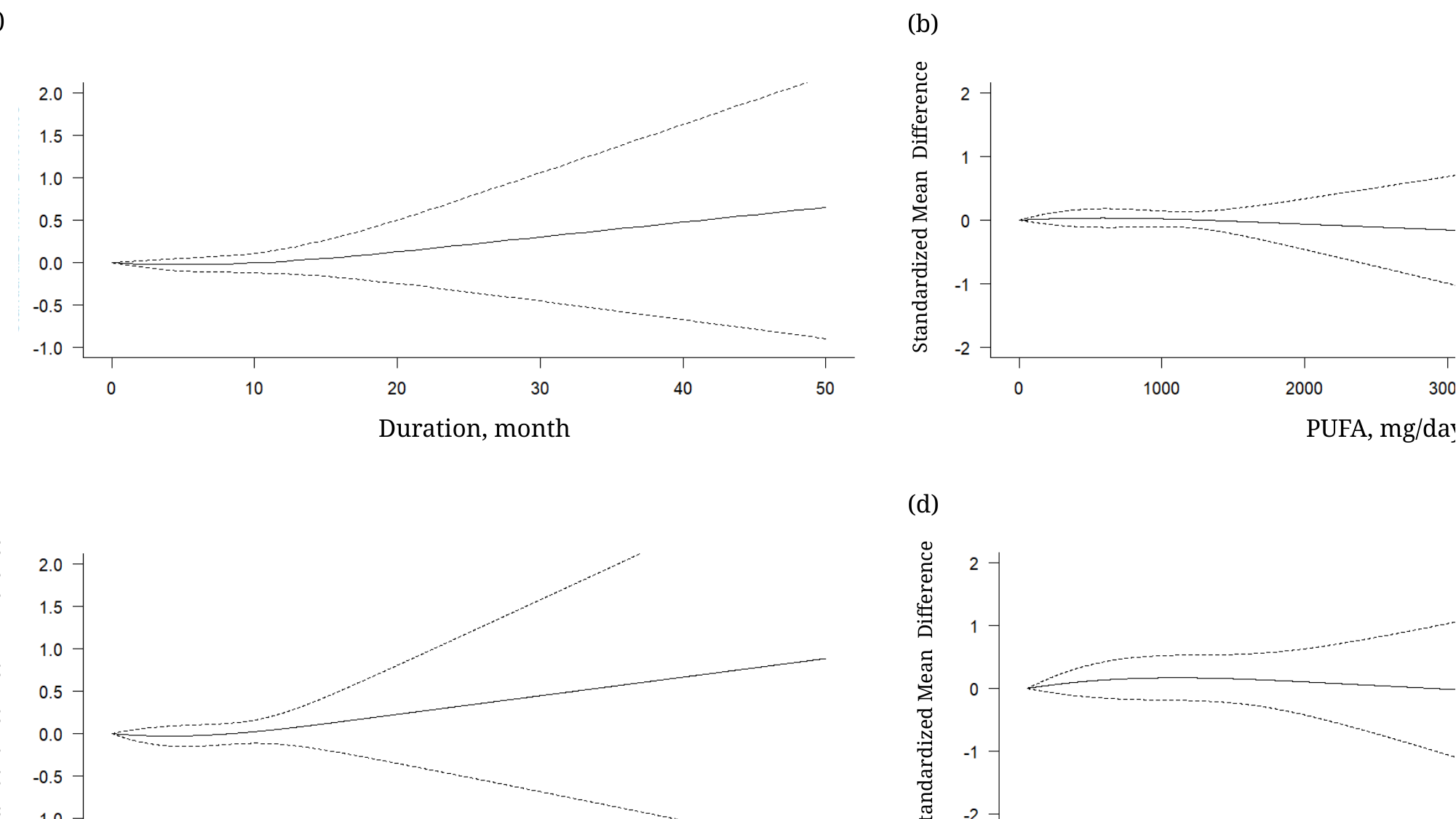

(a)
(b)
Standardized Mean Difference
Standardized Mean Difference
Duration, month
PUFA, mg/day
(c)
(d)
Standardized Mean Difference
Standardized Mean Difference
DHA, mg/day
Total amount of PUFA, g
(e)
(f)
Standardized Mean Difference
Standardized Mean Difference
DHA/EPA ratio
EPA, mg/day

## Slide 7
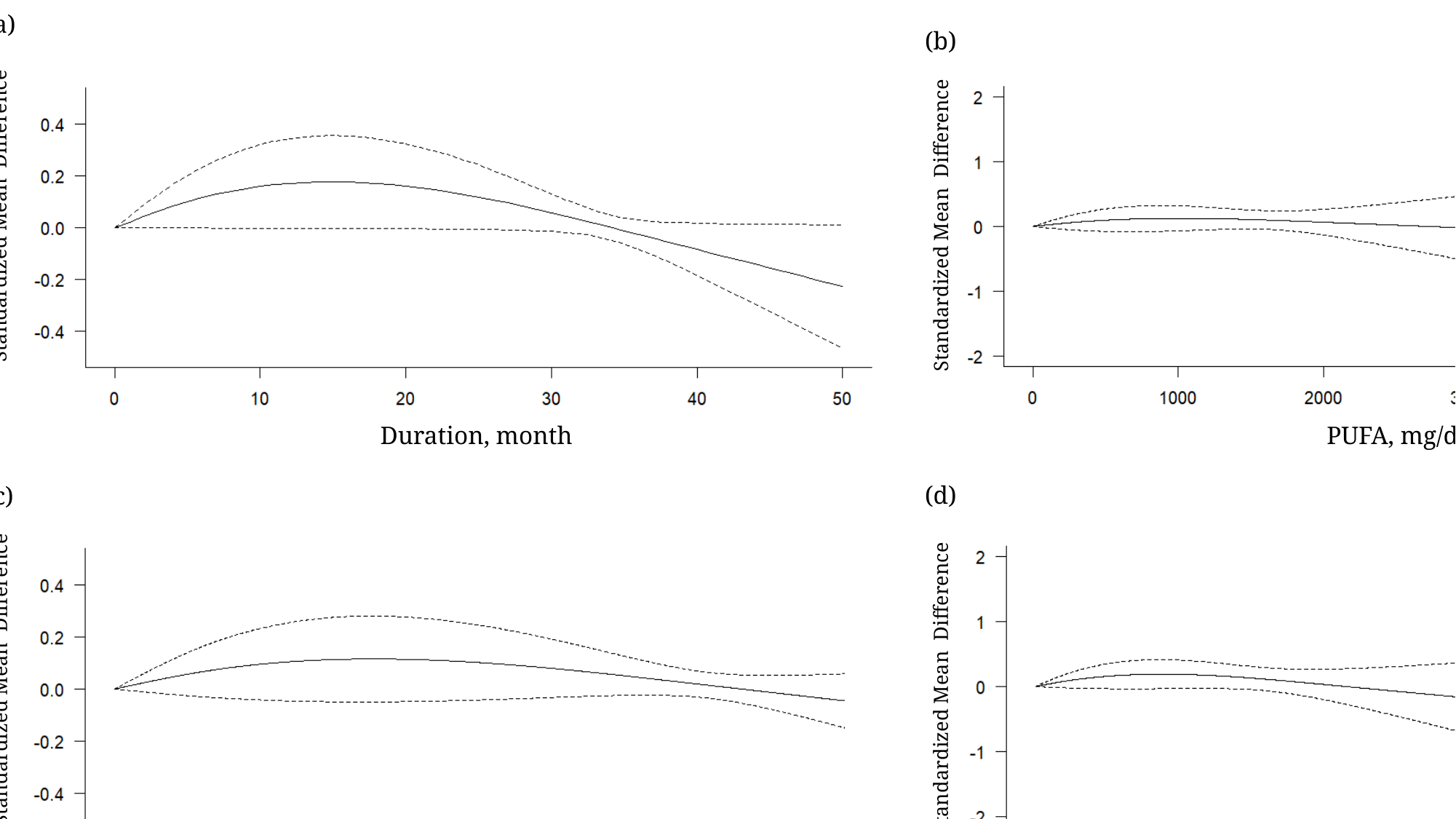

(a)
(b)
Standardized Mean Difference
Standardized Mean Difference
Duration, month
PUFA, mg/day
(d)
(c)
Standardized Mean Difference
Standardized Mean Difference
DHA, mg/day
Total amount of PUFA, g
(e)
(f)
Standardized Mean Difference
Standardized Mean Difference
EPA, mg/day
DHA/EPA ratio

## Slide 8
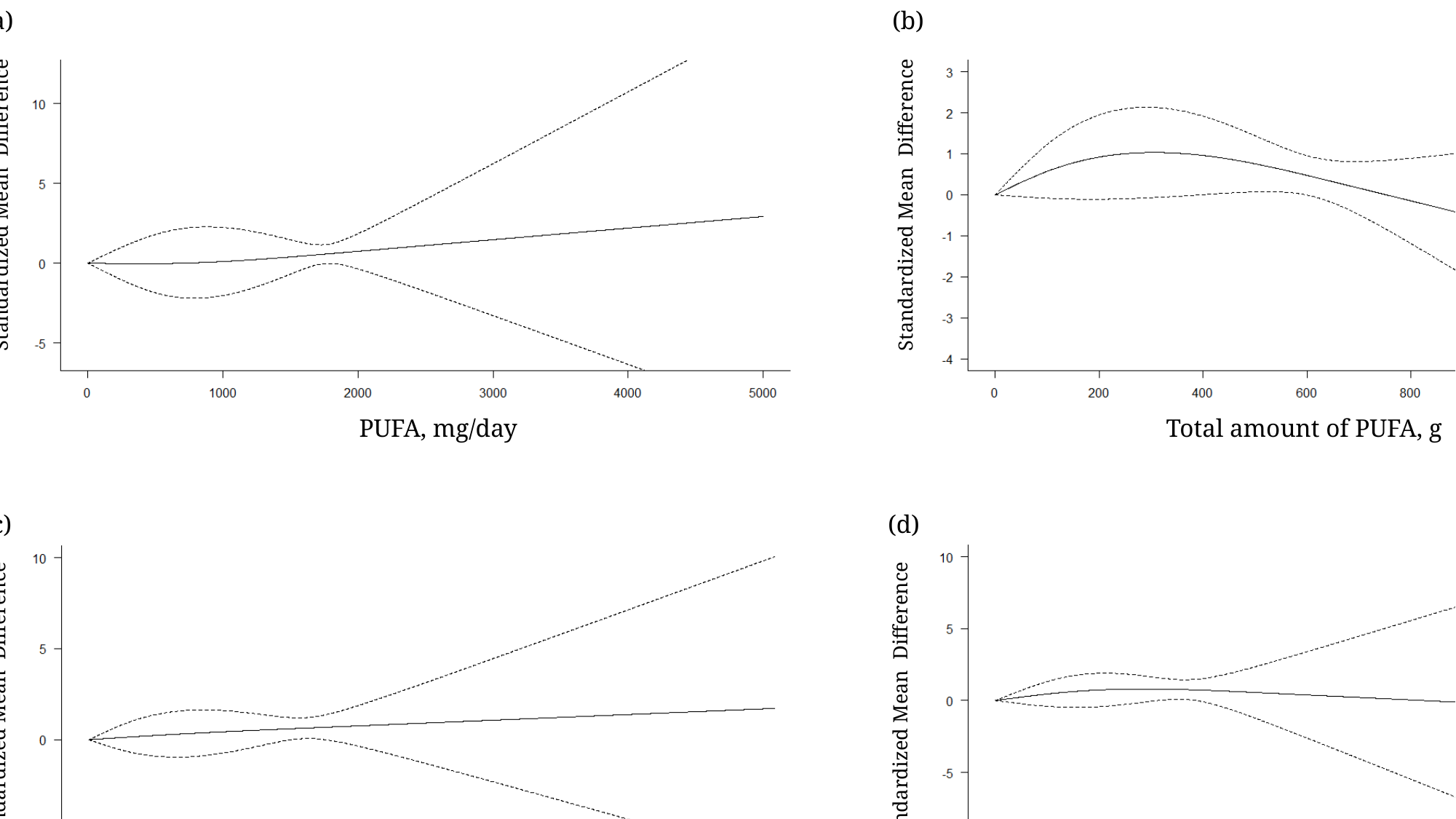

(a)
(b)
Standardized Mean Difference
Standardized Mean Difference
PUFA, mg/day
Total amount of PUFA, g
(d)
(c)
Standardized Mean Difference
Standardized Mean Difference
EPA, mg/day
DHA, mg/day
(e)
Standardized Mean Difference
DHA/EPA ratio

## Slide 9
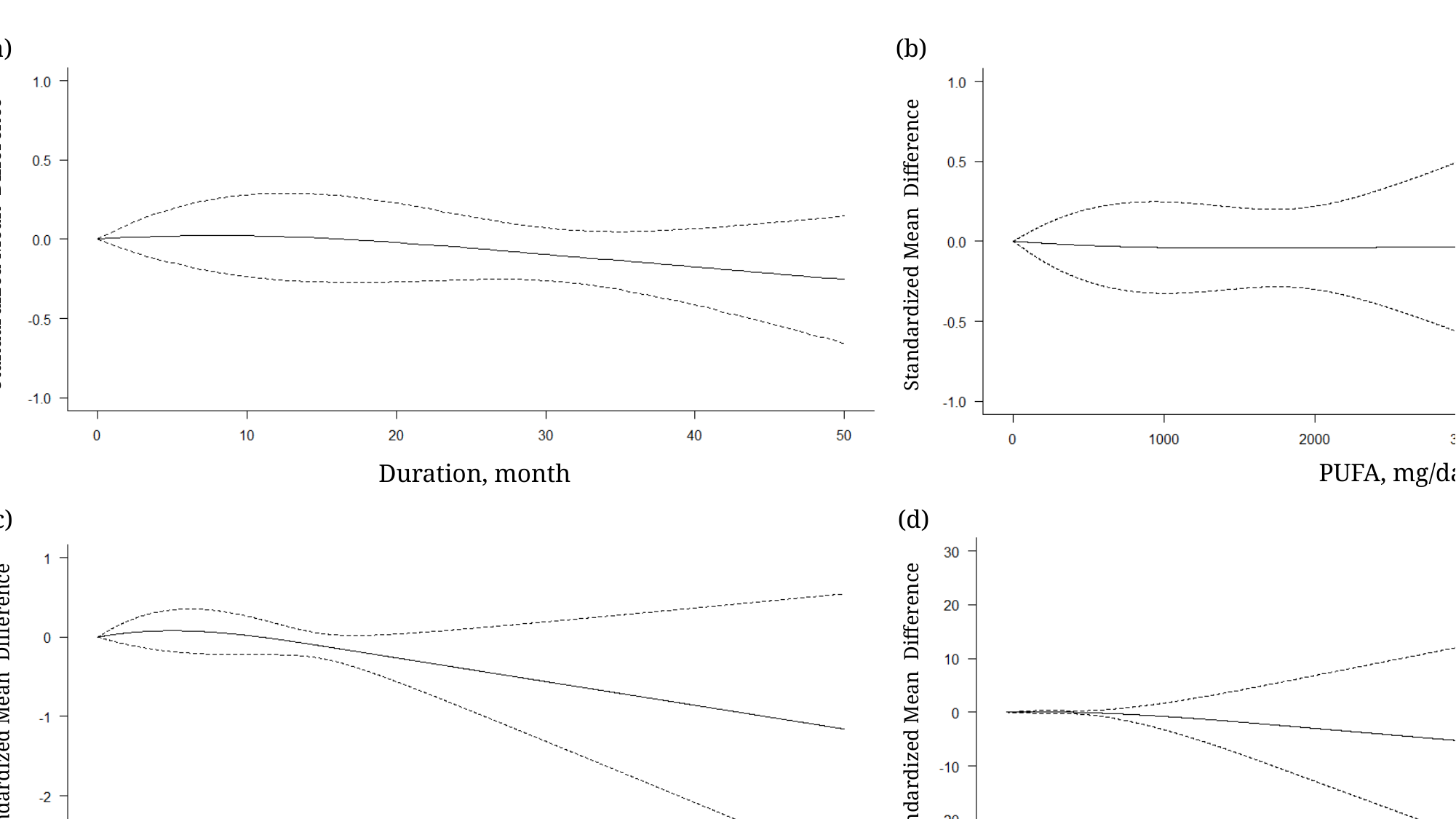

(a)
(b)
Standardized Mean Difference
Standardized Mean Difference
PUFA, mg/day
Duration, month
(c)
(d)
Standardized Mean Difference
Standardized Mean Difference
Total amount of PUFA, g
DHA, mg/day
(e)
(f)
Standardized Mean Difference
Standardized Mean Difference
EPA, mg/day
DHA/EPA ratio

## Slide 10
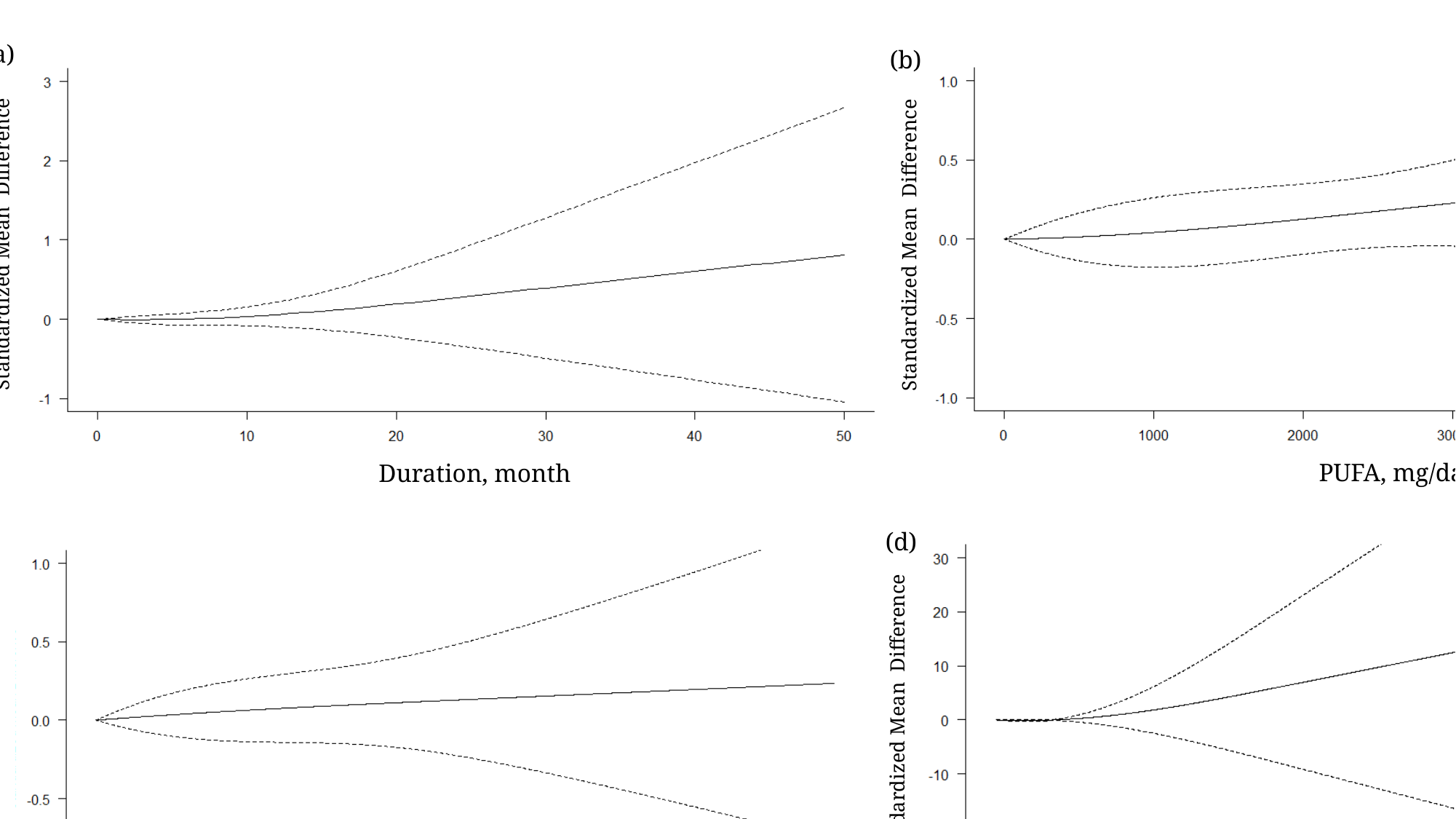

(a)
(b)
Standardized Mean Difference
Standardized Mean Difference
PUFA, mg/day
Duration, month
(d)
(c)
Standardized Mean Difference
Standardized Mean Difference
Total amount of PUFA, g
DHA, mg/day
(f)
(e)
Standardized Mean Difference
Standardized Mean Difference
EPA, mg/day
DHA/EPA ratio

## Slide 11
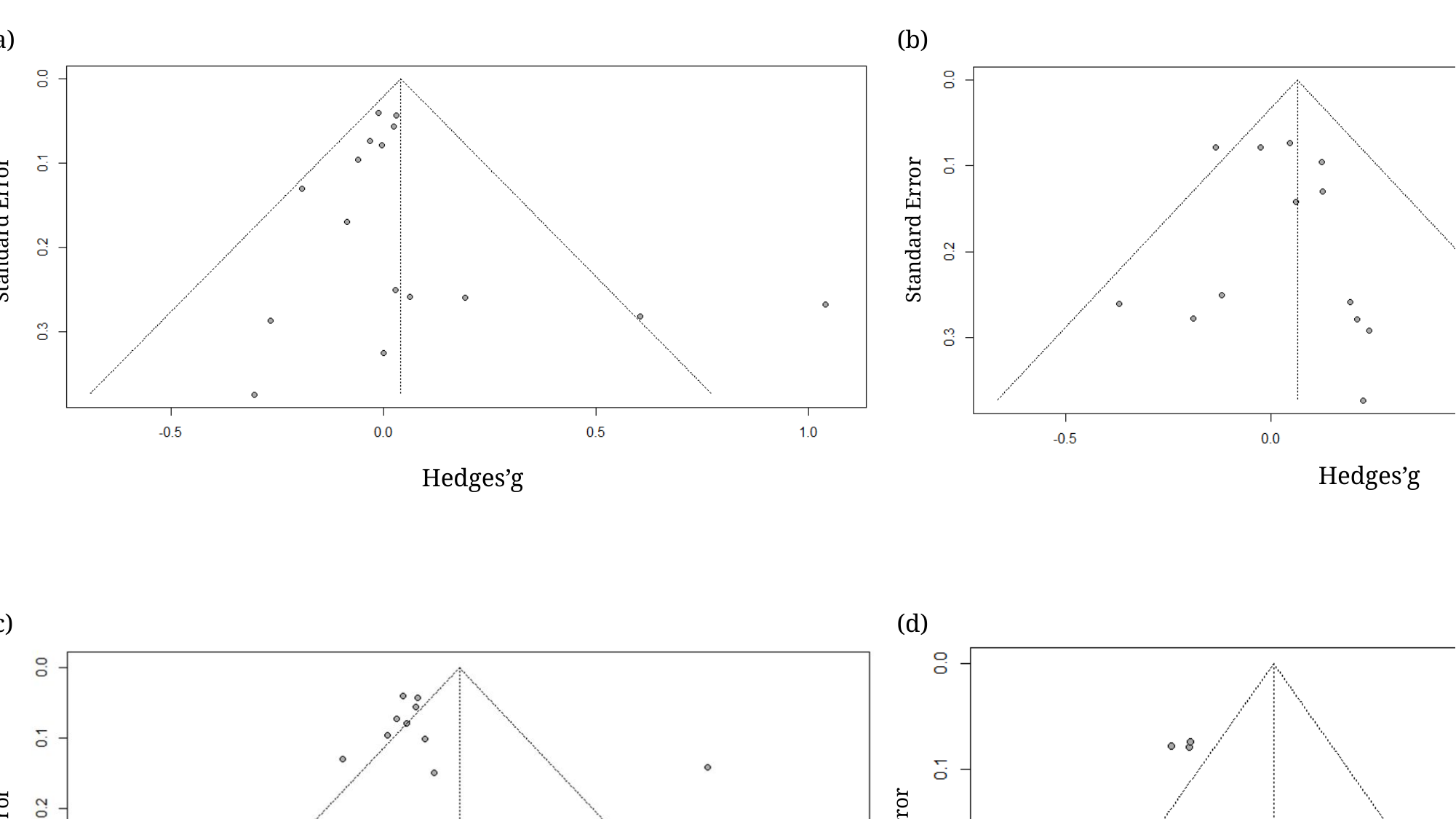

(a)
(b)
Standard Error
Standard Error
Hedges’g
Hedges’g
(c)
(d)
Standard Error
Standard Error
Hedges’g
Hedges’g
(f)
(e)
Standard Error
Standard Error
Hedges’g
Hedges’g
